# Increasing plant protein sources in the diet modulates gut microbiota and tryptophan metabolism in men at cardiometabolic risk

**DOI:** 10.1080/19490976.2026.2677951

**Published:** 2026-05-27

**Authors:** Gaïa Lépine, Anne-Marie Davila, Gwendal Cueff, Gisèle Pickering, Farid Ichou, Caroline Perreau, Catherine Lefranc-Millot, Marine Gilles, Florence Thirion, François Mariotti, Didier Rémond, Hélène Fouillet, Sergio Polakof

**Affiliations:** a Université Clermont Auvergne, INRAE, UNH, Clermont-Ferrand, France; b Université Paris-Saclay, AgroParisTech, INRAE, Palaiseau, France; c Platform of Clinical Investigation Department, University Hospital Clermont-Ferrand, Inserm CIC, Clermont-Ferrand, France; d Foundation for Innovation in Cardiometabolism and Nutrition (IHU ICAN), ICAN Omics, Paris, France; e Roquette Frères, Lestrem, France; f Université Paris-Saclay, INRAE, MGP, Jouy-en-Josas, France

**Keywords:** Plant-based diets, flexitarian diets, legumes, pulses, fibers, indole, indole propionic acid, indoxyl sulfate, kynurenic acid, overweight, obesity, multi-omics

## Abstract

This study investigated the effect of partially substituting dietary animal with plant protein (PP) sources on the fecal microbiota composition and metabolome in men with increased cardiometabolic risk. In a randomized, controlled, crossover feeding trial (NCT04236518), 19 men with high plasma triglycerides and waist circumference completed two 4-week isoenergetic diets: a flexitarian diet high in PP sources (FLEX, 64% PP) and a more animal-based control diet (CON, 36% PP). Fecal microbiota (shotgun metagenomics: taxa and metabolic pathways) and metabolome (targeted LC–MS) profiles were assessed before and after each diet and integrated with the host plasma metabolome. Delta values (Δ_d28-d1_) were computed (*n* = 15 participants with all samples available), inter-individual variation was extracted to account for cross-over design, and OPLS-DA analyses comparing FLEX and CON Δ_d28-d1_ were performed. Variables were selected based on their contribution to the diet discrimination effect (VIP > 1.5) and significant differences between groups (*p*-value < 0.05 from the paired Wilcoxon signed-rank test). The gut microbiota diversity remained unchanged, but FLEX reduced taxa associated with animal-based diets (e.g., *Alistipes putredinis*). Compared to CON, FLEX increased fecal xanthurenic acid and decreased the genetic potential for indole production. Combined with previously reported plasma changes (increased indole propionic acid and decreased indoxyl sulfate after FLEX), these findings suggest a shift away from indole production toward kynurenine and indole propionic acid-related tryptophan pathways, possibly driven by higher fiber intake, particularly from legumes. A one-month flexitarian diet thus modulated in men specific microbial taxa and metabolism, particularly tryptophan catabolism. These coordinated changes in microbial composition, functional potential, and metabolites indicate that diets higher in PP sources influence gut microbiota activities relevant to cardiometabolic health.

## Introduction

Western dietary patterns, characterized by high intakes of red and processed meat, saturated fat, and refined carbohydrates, contribute substantially to the global burden of cardiometabolic diseases.^1^ ^,^ ^2^ Transitioning toward more plant-based diets—high in fruit, vegetables, whole grains, legumes, fiber and phytochemicals—is recognized as key to improve population health while reducing the environmental impact of food systems.^3^ In addition, diets with a higher proportion of plant protein (PP) sources relative to animal protein sources have been associated with better cardiometabolic profiles and lower risks of type-2 diabetes and cardiovascular diseases.^4-6^

Growing evidence indicates that the gut microbiota mediates part of these beneficial effects through diet–microbe–host interactions.^7^ For instance, dietary fiber promotes microbial production of short-chain fatty acids, that enhance gut barrier integrity but also act as key signaling molecules, thereby contributing to the attenuation of systemic inflammation and insulin resistance.^8^ The gut microbiota is also a large contributor to the secondary bile acid pool, which contributes to modulating inflammation,^9^ and specific microbial taxa have been reported to modulate the relationship between fecal bile acids and metabolic risk factors in the context of plant-rich diets.^10^ Polyphenols, high in plant-based diets, are metabolized by gut bacteria into bioactive compounds that have anti-inflammatory and cardioprotective effects.^11^ Conversely, gut microbial metabolism of choline and carnitine—nutrients higher in animal foods—largely contributes to circulating levels of trimethylamine N-oxide, a metabolite associated with increased cardiovascular risk.^12^

PP-rich dietary patterns encompass a wide spectrum, ranging from vegan and vegetarian diets to those that do not exclude meat but where it is largely displaced by plant foods, sometimes refer to as flexitarian diets. However, adherence to restrictive dietary patterns, such as veganism or vegetarianism, remains limited in many populations due to cultural norms, food preferences, and nutritional concerns.^13^ Non-vegetarian diets that emphasize plant-based foods without excluding any animal food category are under scrutiny, as they are increasingly seen as a more pragmatic and actionable strategy for transitioning toward more sustainable diets.

Vegetarian diets have been shown to alter the gut microbiota composition in both observational^14^ ^,^ ^15^ and interventional studies,^16^ although many trials have relied on limited sequencing depth or lacked strict dietary control.^17-19^ In this literature, the gut microbial signature of vegetarian diets has been associated with better cardiometabolic health as compared to omnivorous diets.^15^ The effects of flexitarian diets on the gut microbiota remain poorly characterized. Most previous interventions have examined dietary patterns incorporating commercial plant-based meat substitutes,^20^ ^,^ ^21^ whereas the impact of whole-food–based diets higher in PP sources has been underexplored. To date, no randomized controlled feeding trial has directly compared the effects of a minimally processed, whole-food PP-rich diet with those of a typical Western, animal-based diet on the gut microbiota.

We previously conducted a randomized, controlled and cross-over feeding trial in men with elevated cardiometabolic risk, comparing a flexitarian diet rich in PP sources (64% PP) with an isoenergetic, animal-based control diet (36% PP).^22^ We reported key clinical outcomes and showed that both diets similarly decreased fat mass, insulin resistance (HOMA-IR index) and LDL-cholesterol plasma levels. We further assessed endothelial function (flow-mediated dilatation), protein synthesis and lipid de novo genesis in plasma through isotope tracing, all of which were not modified by the intervention. However, the plasma metabolomic signature was significantly different between diets, with the flexitarian diet modulating branched-chain amino acid-related metabolites toward a more protective profile regarding cardiometabolic risk. Additionally, we reported increased indole propionic acid and decreased indoxyl sulfate plasma levels after the flexitarian diet, comparatively to the control diet, which suggested shifts in gut microbiota activity, especially in relation to tryptophan catabolism pathways.

Here, in this ancillary study, we aimed to extend these findings by characterizing the effects of these two diets on the gut microbiota composition and metabolic activity using fecal shotgun metagenomic sequencing and targeted metabolomics. We further integrate these novel data with previously acquired plasma metabolome profiles to explore host–microbiota metabolic interactions. We hypothesized that the partial replacement of animals with plant protein sources would modify the gut microbiota composition and shift microbial and host metabolic pathways related to tryptophan metabolism.

## Materials and methods

### Study design

The participants and study design have been described in detail elsewhere.^22^ This study was authorized by an ethical committee (the Committee for the Protection of Individuals Sud-Mediterranee I, France) in 2019 (RBHP 2019 PICKERING 2) and registered at the French National Agency for the Safety of Medicines and Healthcare Products (2019-A02447-50) and clinicaltrials.gov (NCT04236518). Briefly, 19 adult men (25–55 y) with overweight or obesity (BMI 25–35 kg/m²), enlarged waist circumference (>94 cm), and at least one of the following conditions were included: elevated fasting plasma triglycerides (>1.49 g/L); elevated fasting glucose (≥5.6 mmol/L); low fasting HDL-cholesterol (<1.03 mmol/L); and elevated systolic (≥130 mmHg) or diastolic (≥85 mmHg) blood pressure. Only men were included to reduce population heterogeneity, and because some of the outcomes studied here are known to be influenced by the menstrual cycle phase.^22^ ^,^ ^23^

In this monocentric, randomized, cross-over feeding trial, participants followed two isocaloric diets for one month each: (1) a flexitarian diet high in PP sources (PP: 63.9% of total protein intake, FLEX diet), including all food groups but limited portions of meat and fish and emphasizing minimally processed, protein-rich plant foods—particularly legumes—with minimal consumption of meat substitutes; and (2) a control diet predominantly based on animal protein sources (PP: 35.5% of total protein intake, CON diet). The control diet was designed to mimic the proportion of animal protein typically consumed in Western countries which isaround 65%,^24^ ^,^ ^25^ in line with the usual intake of our study population (64%).^22^

Diets were provided as ready-to-eat lunches and dinners, along with specific dietary recommendations for breakfast and snacks. Compliance was monitored by checking daily food intake reports and by assessing ^15^N enrichment ratio of plasma protein, reflecting animal product intake. A two-week wash-out period, during which participants resumed their habitual diet without specific dietary constraints, was implemented between diets.

Stool samples were collected during visits at the clinical center on the mornings of the first (d1) and last day (d28) of each dietary phase, following the procedures of the International Human Microbiome Standards. A 1 mL aliquot was collected according to SOP4,^26^ and a 500 µL aliquot was collected in stabilization solution (RNAlater, R0901, Sigma) according to SOP5.^27^ Participants were instructed to collect the stool sample on the morning of the visit or the preceding day and to store it in a cooler until the visit. Upon reception at the clinical center, all samples were immediately stored at −80 °C until further analyses.

### Analyses

The first step of the fecal metagenomic analyses, DNA extraction from collected stool samples, was carried out by the SAMBO platform (MetaGenoPolis, INRAe Jouy-en-Josas, France). After manual aliquoting, the samples were centrifuged to remove the preservation solution. The samples were then processed following a protocol adapted from MGP SOP 000 V1 [https://mgps.eu/sops/mgp-sop-001-v1/, originally developed for saliva samples but applicable to stool samples https://www.protocols.io/view/protocol-for-dna-extraction-from-saliva-samples-us-dm6gpjm11gzp/v1] for the QIAGEN DSP Virus/Pathogen Kit (Qiagen, Hilden, Germany): 250 µL of guanidium thiocyanate, 40 µL N-lauroyl sarcosine (10% solution), and 500 µL N-lauroyl sarcosine (5% solution in 1 × PBS) were added to each frozen pellet, which were then homogenized with a toothpick and vortexed. Following this, 500 µL of 0.1 mm glass beads (dry, not in suspension) were added to the samples. They were subsequently incubated at 70 °C in a thermomixer for one hour, with stirring at 1400 rpm. After centrifugation at 3486 × *g* for five minutes, the lysate was collected in a new tube. The pellet was then washed with 500 µL of TENP (50 mM Tris-HCL, 20 mM EDTA, 10 mM NaCl, saturated with PVPP). The tube was vortexed and centrifuged at 3486 × *g* for five minutes, after which the recovered lysate was pooled with the previous one. The lysate was then centrifuged for 10 min at 3486 × *g*, after which 800 µL were transferred to a new tube. The resulting sample was used for purification with magnetic beads on the QIASymphony platform (Qiagen, Hilden, Germany).

Fragment Analyzer (Agilent Technologies, Santa Clara, US) using the Genomic 50 kb Kit. 500 ng of DNA was fragmented by sonication using the E220 focused ultrasonicator (Covaris, Woburn, US) and then underwent double purification using the DNA Clean Beads Kit (MGI Technology, Shenzhen, China) to select fragment sizes around 400 bp. 100 ng of sized DNA was used to construct libraries using the Universal Library Prep Kit and Universal Barcode Set (MGI Technology, Shenzhen, China). The amplified libraries (8 PCR cycles) were checked with the HS Small Fragment Kit on a Fragment Analyzer (Agilent Technologies, Santa Clara, US) before being circularized and pooled. 50pM of the circularized and pooled libraries were used to make DNA nanoball (DNB), which were loaded onto FCL (Flow Cell Large) flow cells and sequenced with the high-throughput sequencing kit PE150 (MGI Technology, Shenzhen, China) on a DNBseq G400RS platform (MGI Technology, Shenzhen, China) to obtain a minimum of 20 million paired-end reads per sample. Using fastp,^28^ low-quality reads were trimmed or filtered out. The remaining high-quality reads successfully mapped to the human reference genome (T2T-CHM13v2.0) using bowtie2^29^ were further discarded. To account for differences in sequencing depth, 20 million read pairs (i.e., 40 million of single reads) were randomly selected for each sample with seqtk (https://github.com/lh3/seqtk). Then, species and functional modules abundance tables were generated with Meteor2^30^ (version 2.0.14, normalization = fpkm). Given evidence of microbial transmission between the oral cavity and the gut in both health and disease,^31^ ^,^ ^32^ stool-derived DNA sequences were profiled against both the “human_gut” and the “human_oral” references available through Meteor2, and the resulting abundance tables were subsequently merged. In short, references consisting of microbial gene catalogues (10.4 million for the human gut microbiome^33^ and 8.4 million for the human oral microbiome)^34^ were clustered into 1990 and 853 Metagenomic Species (MSP) with MSPminer,^35^ respectively. Gene catalogues are also functionally annotated with KEGG (v107)^36^ to infer KEGG modules, Gut-Metabolic Modules (GMM)^37^ and Gut-Brain Modules (GBM)^38^ potential. All the abundance tables are computed as previously described.^30^ The gut microbiota richness (or MSP richness) was computed as the number of MSP detected in a given sample (i.e., MSP whose abundance is not null). Oral richness is defined as the number of MSP whose usual environment is the oral cavity; the oral richness ratio is the ratio between oral richness and total MSP richness. The Shannon diversity index was computed with the package vegan (v2.6–10). Quality control was performed using Crocodeel v1.0.3^39^ (https://github.com/metagenopolis/CroCoDeEL).

For the fecal metabolomic analysis, feces (100 mg) were homogenized in 1 mL of ultrapure water using a Precellys Evolution instrument (Bertin Instruments, Montigny-le-Bretonneux, France), and the supernatant was collected (100 µL) and mixed with frozen acetonitrile (VWR International) and internal standards, which consisted in a mix of 16 amino acids labeled in ^13^C and ^15^N (algal amino acid mixture-13C, 15N, Sigma-Aldrich), before being vortexed and sonicated. The samples were centrifuged (2 min, 10,000 × *g*, 4 °C), incubated at 4 °C during 1 h for slow protein precipitation process, centrifuged again (15 min, 20,000 × *g*, 4°). The supernatants were transferred, dried and stored at −80 °C prior to analyses. Pellets were diluted 3-fold and reconstituted in H_2_O/ACN (20/80) just before analyses. The fecal metabolome was measured by LC-HRMS using UPLC Waters Acquity (Waters Corp.) with HILIC chromatographic column (Sequant ZIC-pHILIC column 5 µm, 2.1 × 150 mm, Merck) and a Q-Exactive mass spectrometer (Thermo Scientific). Experimental settings for metabolomic global approaches by LC-HRMS were carried out as detailed previously.^40^ LC-MS raw data were first converted into mzXML format using the MSconvert tool.^41^ Peak detection, correction, alignment and integration were processed using the XCMS R package with the CentWave algorithm.^42^ ^,^ ^43^ The resulting dataset was Log-10 normalized, filtered and cleaned based on quality control samples as described.^44^ Metabolite abundance was normalized by fecal water content. The remaining metabolomic features were annotated based on their mass over charge ratio (m/z) and retention time using an “in-house” database detailed in.^1^ The metabolomic features were also putatively annotated based solely on their m/z using public databases such as the human metabolome database HMDB^45^ and the Kyoto Encyclopedia of Genes and Genomes database (KEGG).^46^ The analysis was targeted on a panel of metabolites involved in tryptophan–kynurenine, amino acids and choline metabolism, overall 74 metabolites were fully identified (level 1) and 84 were putatively identified (level 2) according to international identification standards.^47^

To measure short-chain fatty acid (SCFA) fecal concentrations, fecal samples (100 mg) were homogenized in 500 µL of NaOH solution (0.005 mol/L, Sigma-Aldrich), including internal standards (acetate-D3, butyrate propionate-D2 and valerate-D9, Sigma-Aldrich). The supernatants were collected, mixed with 500 µL of a propanol/pyridine mix (3:2 v/v, Sigma-Aldrich) and vortexed. SCFA were derivatized using isopropyl chloroformate (Sigma-Aldrich) iPCF and extracted in 0.5 mL of hexane (Sigma-Aldrich). SCFAs were quantified by gas chromatographic/mass spectrometry using an ISQ LT™ equipped with a Triplus RSH (Thermo Fisher Scientific, Illkirch, France) and a fused-silica capillary column with a (5%-phenyl)-methylpolysiloxane phase (DB-5ms, J&W Scientific, Agilent Technologies Inc., USA) of 50 m × 0.25 mm i.d coated with 0.25 µm film thickness. Data processing was performed using Xcalibur® software (version 3.0; Thermo Fisher Scientific). Quantification was done on a single ion monitoring using Electron impact ionization, and concentration of SCFA were calculated based on a calibration curve and by normalizing analyte peak areas to the corresponding internal standard peak areas.

### Statistical methods

All analyses were performed using R software (version 4.4.1). The effect of the time (d1 vs d28), diet (FLEX vs CON), sequence (FLEX-CON vs CON-FLEX) and associated interaction were assessed on alpha diversity metrics and short-chain fatty acid values using the Wald test statistics from the nonparametric Analysis of longitudinal data in factorial experiments (nparLD package, version 2.2).^48^

The effect of the intervention on microbiota β-diversity was assessed in a subset of participants with all timepoints available. Bray‒Curtis dissimilarity and associated permutational multivariate analysis of variance (PERMANOVA) were computed with the R package vegan (v2.6-10) on MSP abundance. Principal coordinate analysis (PCoA) was performed with the R package ade4 (v1.7.23).

Before performing multivariate analyses to test the effect of diets on the fecal metagenomics and metabolomics datasets, different data transformation steps were performed. Owing to high inter-individual and intra-individual variability in fecal microbiota composition and the presence of low-abundance species that are not always detected, the delta metagenomics matrix was highly sparse. Only metagenomics with a prevalence across all samples above 25% were selected for further analyses. Delta values (Δ_d28-d1_) were computed as (d28 value—d1 value) for each dataset. To account for the cross-over design with two delta values per individuals we extracted the intra-individual variation (WithinVariation function, MixOmics R package, v 6.30.0) on a subset of participants with all timepoints available. The resulting fecal metagenomics and metabolomics data matrix of intra-individual variations between the CON-delta and FLEX-delta values were mean-centered and unit-variance scaled before being analyzed by orthogonal-partial least square-discriminant analysis (OPLS-DA) to explore the effect of diet (ropls R package, v1.38.0). Model prediction ability and fit were assessed by R2Y and Q2Y values, respectively, and associated *p*-values were calculated by permutation test (*n* = 100 repetition). Variables contributing strongly to the diet effect were selected based on Variable Importance in the projection (VIP) > 1.5. On this subset of variables, the diet effect was further assessed by paired Wilcoxon signed-rank on delta values (*p*-value < 0.05) (Rstatix R package, v0.7.2).

The gut microbiota composition and activity potential are tightly intricated with host physiological state. To account for these intercorrelations and explore the associations between these different biological levels, we performed a multi-omics data integration including fecal metagenomics, fecal metabolomics and plasma metabolomics (derived from a previously described untargeted LC‒MS/MS analysis^22^ ) based on Δ_d28-d1_ calculated as described above. In the metagenomic dataset, only MSP with a prevalence >25% were retained (as described above). In the plasma metabolomics dataset, only metabolite with a validated annotation (*n* = 158) were included. The analysis was conducted in samples with available data from the 3 datasets. Each dataset was transformed to extract the intra-individual variation (WithinVariation() function, MixOmics R package), and the resulting data matrix were mean-centered and unit-variance scaled before being analyzed by multi-bloc partial least square-discriminant analysis (mixOmics R package, block.plsda() function) to explore the effect of the diet. Model performance was assessed by cross-validation (10 folds), with 100 repetitions. Sample class (FLEX or CON) were predicted using the maximum distance in each omic dataset, and predictions were combined by weighted vote, where more importance was given to predictions related to the omics dataset most correlated with the components associated with diet discrimination. From these results, the misclassification error rate was calculated across all samples.^49^

To explore further the relation between omics datasets, we extracted a bipartite relevance network from the multi-bloc PLS-DA model obtained in the initial analysis, similar to a correlation network but representing only associations between pairs of variables belonging to different omics datasets (mixOmics R package, network() function).^49^ Associations with a similarity coefficient (equivalent to a robust approximation of Pearson correlation) above 0.5 were included in the final network. This threshold was set by analogy with correlation coefficient thresholds^50^ to exclude weak correlations and increase the chance of observing meaningful biological associations. A subnetwork was extracted to represent only the association between variables with a diet effect, as assessed by mono-omics analysis. The fecal species and metabolites included had a VIP > 1.5 in OPLS-DA model testing diet effect on Δ_d28-d1_ values. The plasma metabolites included had a loading score on the diet component > 0.01 in linear model-principal component analysis (LIMPCA) and a significant diet effect when individually assessed by a linear mixed model test (*p*-diet < 0.05), see^22^ for details.

All other figures were drawn with the R packages ggplot2 (v4.0.0) and ggpubr (v0.6.0).

## Results

### Baseline characteristics

As previously reported,^22^ the 19 participants who completed the study were middle aged (41.7 ± 7.1 y) males, with overweight (BMI: 28.4 ± 2.4 kg/m²), enlarged waist circumference (102.7 ± 5.9 cm) and elevated fasting plasma triglycerides (2.1 ± 0.7 g/L). During the CON intervention, total protein intake accounted for 16.3% of total energy intake (TEI), with 35.5% derived from PP sources, values comparable to baseline intakes (protein: 16.4 ± 2.3% TEI, PP: 36.0 ± 8.0%). In contrast, the FLEX intervention resulted in a slightly lower protein intake (13.3 ± 0.8% TEI) and nearly reversed the PP:AP ratio in the diet (63.9 ± 4.5%). Detailed dietary data and clinical outcomes at baseline and after each intervention are reported elsewhere.^22^

A few fecal samples were missing for some participants at specific time points. During the CON intervention, all samples were collected at d1, but one sample was missing at d28, whereas during the FLEX intervention, one sample was missing at d1 and three at d28.

A total of 1234 microbial species were detected, of which 425 showed a prevalence greater than 25% across all samples. The distributions of the phyla and top 20 most abundant MSP across individuals and timepoints are presented in Supplemental Figures S1 and S2, illustrating large inter-individual variability.

Neither alpha diversity (assessed by MSP richness) nor beta diversity (assessed by principal coordinate analysis) was different at baseline across sequence groups (CON followed by FLEX, or the reverse), as shown in Supplemental Figure S3.

### Increasing PP sources do not impact fecal microbiota diversity but shifted specific microbial taxa and metabolic pathways

**Figure 1.**
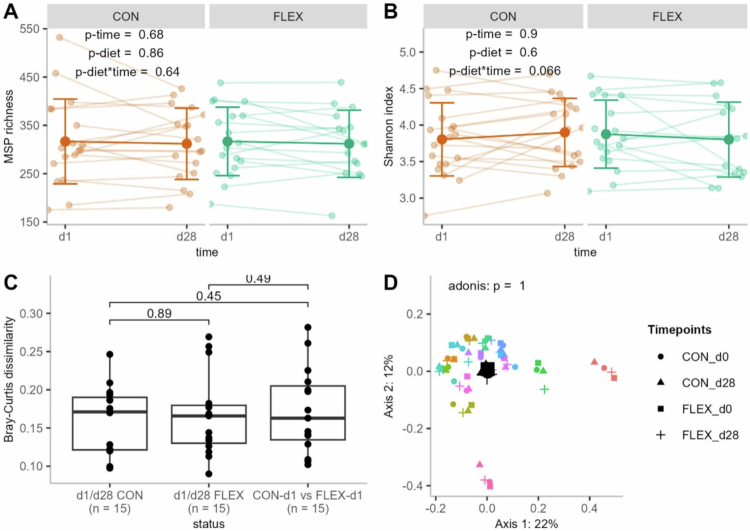
Effect of the flexitarian (FLEX) and control (CON) diets on fecal metagenomic alpha and beta diversity metrics. (A) Metagenomic species (MSP) richness (*n* = 19) and (B) Shannon index before (d1) and after (d28) each diet (*n* = 19). (C) Bray‒Curtis dissimilarity across conditions (*n* = 15). (D) Principal coordinates analysis (PCoA) based on log10-transformed MSP relative abundance using Bray–Curtis distances, with the diet effect assessed by adonis test (*n* = 15). Small points represent individual samples, and large points indicate group centroids. The values are mean and SD (panel A and B), or median and quartiles (panel C).

Alpha diversity metrics showed no changes following the intervention, with no effect of time, diet, or their interaction on total MSP richness and Shannon index (Figure 1A and B). Beta diversity, assessed by Bray–Curtis dissimilarity, also remained unchanged throughout the intervention (Figure 1C and D).

Fecal metagenomics data (*n* = 15 participants with all 4 timepoints) were included in the subsequent multivariate analyses integrating fecal species, metabolic pathways, and metabolites datasets. After extracting intra-individual variability (see Supplemental Figure S4) to account for the cross-over design, an OPLS-DA model was applied to Δ_d28-d1_ values of MSP relative abundances. The resulting model showed excellent fit (R^2^Y = 0.989, permutation test *n* = 100: *p*-value = 0.01) and strong predictive ability (Q^2^Y = 0.91, permutation test *n* = 100: *p*-value = 0.01), effectively discriminating the effects of the two diets (see Supplemental Figure S5).

**Figure 2.**
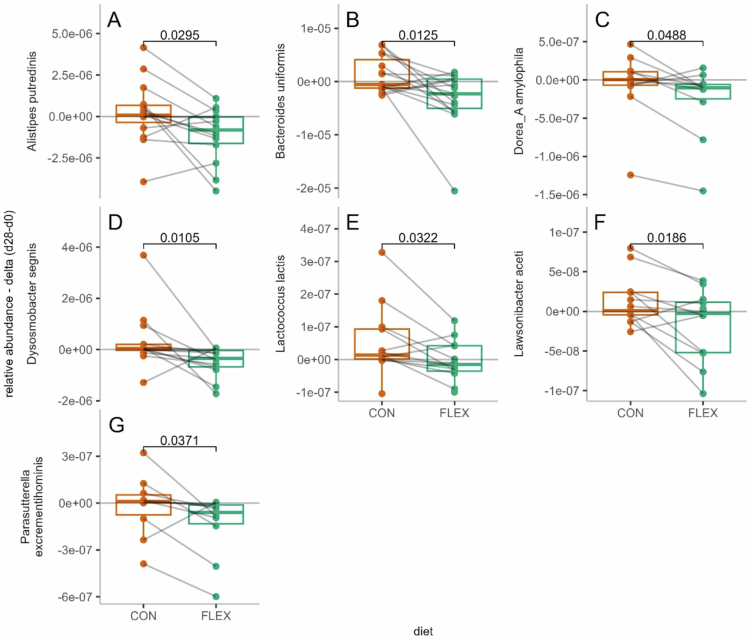
Fecal metagenomic species with differential relative abundance delta values (Δ_d28-d1_) after the flexitarian (FLEX) and control (CON) diets. Displayed species had variable importance in the projection (VIP) scores > 1.5 and significantly different relative abundance changes between diets, as assessed by paired Wilcoxon signed-rank tests (*p* < 0.05). The values are presented as medians with 1st and 3rd quartiles. (A) Alistipes putredinis; (B) Bacteroides uniformis; (C) Dorea_A amylophilia; (D) Dysosmobacter segnis; (E) Lactococcus lactis; (F) Lawsonibacter aceti; (G) Parasutterella excrementihominis

Twenty-eight MSP contributed highly to diet discrimination (VIP > 1.5) (Supplemental Table S1), and 7 showed significantly different Δ_d28-d1_ values between CON and FLEX. These included *Dorea A amylophila, Alistipes putredinis, Lactococcus lactis, Dysmobacter segnis, Parasutterella excrementihominis, Lawsonibacter aceti*, and *Bacteroides uniformis* (Figure 2).

We then explored the impact of diets on the metagenomic functional repertoire. After extracting intra-individual variability (see Supplemental Figure S6), an OPLS-DA model was applied to the Δ_d28-d1_ values of pathway relative abundances. The resulting model showed excellent fit (R^2^Y = 0.984, permutation test *n* = 100: *p*-value = 0.01) and strong predictive ability (Q^2^Y = 0.944, permutation test *n* = 100: *p*-value = 0.01), effectively discriminating between diets (see Supplemental Figure S7).

**Figure 3.**
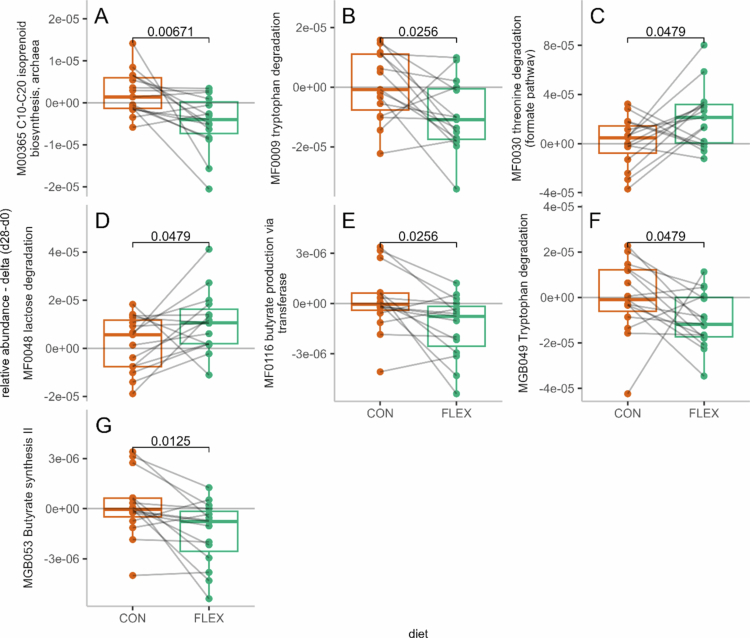
Fecal metagenomic pathways with differential relative abundance Δ_d28-d1_ value after the flexitarian (FLEX) and control (CON) diets. Displayed pathways had variable importance in projection (VIP) scores > 1.5 and significantly different Δ_d28-d1_ values between diets, as determined by paired Wilcoxon signed-rank tests (*p* < 0.05). (A) M00365 – C10–C20 isoprenoid biosynthesis, archaea; (B) MF0009 – tryptophan degradation; (C) MF0030 – threonine degradation (formate pathway); (D) MF0048 – lactose degradation; (E) MF0116 – butyrate production via transferase; (F) MGB049 – tryptophan degradation; (G) MGB053 – butyrate synthesis II.

Twenty-four pathways contributed highly to diet discrimination (VIP > 1.5), and 6 showed significantly different Δ_d28-d1_ values between CON and FLEX. These pathways were associated with butyrate production, amino acids degradation (including tryptophan and threonine degradation), lactose degradation, and isoprenoid synthesis (Supplemental Table S2, Figure 3).

### While SCFA levels were not altered, the global fecal metabolome signature was modified by experimental diets

Regarding the SCFA results (*n* = 19 participants), the diet sequence (order of FLEX and CON consumption) significantly affected the fecal acetate, propionate, valerate and total SCFAs levels, and its interaction with time was significant for all SCFAs (Supplemental Table S3). However, no significant time*diet interaction was observed between diets. Pairwise comparisons stratified by sequence order showed no differences between diets at any timepoint for all SCFAs, except for valerate at d28 in the CON-FLEX sequence (see Supplemental Figure S8).

Fecal metabolomic profiling quantified 158 metabolites, including 74 fully identified (level 1) and 84 putatively identified (level 2) compounds, in accordance with international metabolite identification standards.^47^

After extracting intra-individual variability (see Supplemental Figure S9), an OPLS-DA model was applied to the Δ_d28-d1_ values of fecal metabolite relative abundance (*n* = 15 participants with all samples available). The model showed a strong fit (R^2^Y = 0.988, permutation test *n* = 100: *p*-value = 0.01) and predictive ability (Q^2^Y = 0.959, permutation test *n* = 100: *p*-value = 0.01), clearly discriminating between the two diets (see Supplemental Figure S10).

**Figure 4.**
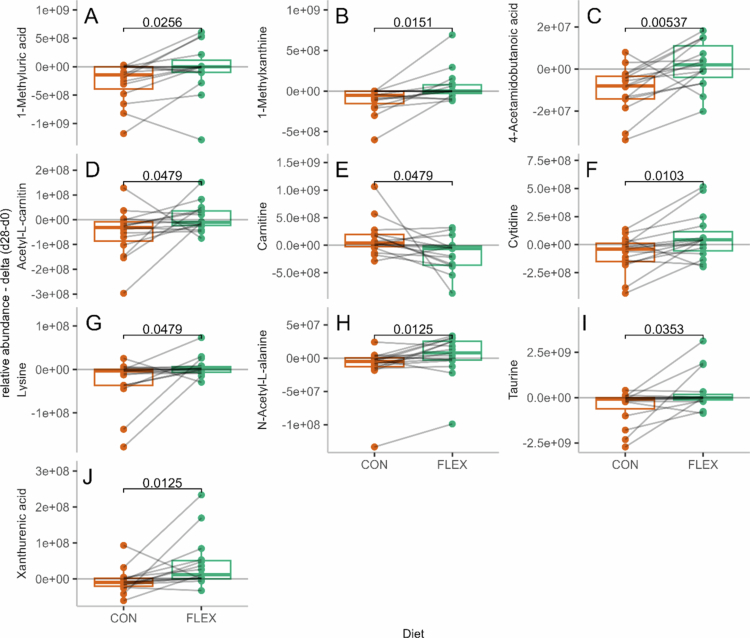
Fecal metabolites relative abundance Δ_d28-d1_ values after the flexitarian (FLEX) and control (CON) diets. Presented metabolites had a variable importance in the projection (VIP) > 1.5, and their Δ_d28-d1_ values differed significantly between the diets, as assessed by paired Wilcoxon signed-rank tests (*p* < 0.05). The values are presented as medians with 1st and 3rd quartiles. (A) 1-Methyluric acid; (B) 1-Methylxanthine; (C) 4-Acetamidobutanoic acid; (D) Acetyl-L-carnitine; (E) Carnitine; (F) Cytidine; (G) Lysine; (H) N-Acetyl-L-alanine; (I) Taurine; (J) Xanthurenic acid

Nineteen metabolites contributed highly to diet discrimination (VIP > 1.5), and 10 showed significantly different Δ_d28-d1_ values between CON and FLEX. These included metabolites related to amino acid metabolism (4-acetamidobutanoic acid, taurine, carnitine, lysine, acetyl-carnitine, N-acetyl-L-alanine), purine metabolism (1-methylxanthine, cytidine, 1-methyluric acid), and kynurenine metabolism (xanthurenic acid) (Supplemental Table S4, Figure 4).

### Integrating host physiology with fecal microbiota through multi-omics analysis

Fifteen participants had fecal specie, fecal metabolite and plasma metabolite data available at all 4 time points and were included in the multi-block PLS-DA analysis. The integrated datasets were strongly correlated, with correlation coefficients of 0.92 and 0.84 between plasma or fecal metabolites and fecal species and 0.84 between plasma and fecal metabolites. Sample separation according to each dataset and key features contributing to diet discrimination are presented in Supplemental Figures S11 and S12. The overall weighted-vote misclassification error rate of the integrative model was 33%. Given its relatively lower predictive performance compared with the single omics analyses, features contributing to diet discrimination are not further discussed.

**Figure 5.**
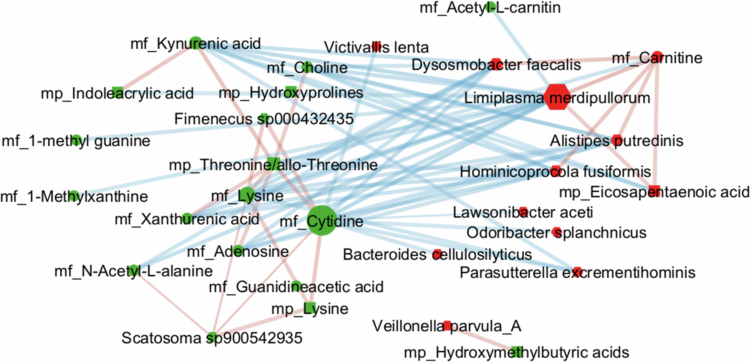
Bipartite relevance network derived from multi-block PLS-DA analysis, illustrating inter-omics relationships among selected fecal species, fecal metabolites, and plasma metabolites contributing to the diet effect. Fecal species and metabolites included had a variable importance in the projection (VIP) score > 1.5 in OPLS-DA models applied to Δ_d28-d1_ values after the flexitarian (FLEX) and control (CON) diets. The plasma metabolites had a diet-loading > 0.01 in the LIM-PCA model and showed a significant diet effect according to linear mixed models (*p*-diet < 0.05) (see^22^ Edges width is proportional to the similarity coefficient (a correlation-like measure)); only associations with similarity > 0.5 are displayed. The edge color indicates the direction of the association (red, positive; blue, negative). The node color indicates the direction of the diet-induced change (green, Δ_d28-d1_ FLEX > Δ_d28-d1_ CON; red, Δ_d28-d1_ FLEX < Δ_d28-d1_ CON). The node size is proportional to the betweenness centrality.

On the other hand, a relevance network was computed to visualize correlation-like associations (similarity coefficient >0.5) between pairs of variables from different datasets (Supplemental Figure S13), and a subnetwork restricted to features previously identified as contributing to diet discrimination as assessed by previous single omics analysis was extracted (Figure 5). Within this subnetwork, the bacterial species *Limiplasma merdipullorum* and the fecal metabolite cytidine emerged as highly central nodes, each connected to variables from all three omics datasets.

## Discussion

In men at elevated cardiometabolic risk, a one-month exposure to a flexitarian diet higher in PP sources, compared with a more animal-based control diet, did not shift fecal microbiome diversity but significantly modulated several microbial species, microbial metabolic pathways, and fecal metabolites. These findings, combined with previously described changes in plasma metabolites, suggest that gut microbial tryptophan degradation pathways were differentially modulated by the intervention diets. This works adds a new line of evidence regarding the impact of diets rich in PP source, provided as a whole diet with minimally processed PP sources, on gut microbiota composition and activity through a cross-over trial with tight dietary control in a context where interventional trials are scarce.

### Microbial signatures reflect reduced animal protein intake

In the present work, neither the alpha nor beta diversity of the fecal microbiome was significantly affected by nutritional intervention. Among the bacterial species that contributed to the effects of the diet, several have previously been associated with animal-based diets. *A. putredinis*, a Gram-negative, proteolytic, and bile-tolerant bacterium,^51^ ^,^ ^52^ decreased after the flexitarian diet in our study. This finding is consistent with previous reports showing an increase in these species after a 5-d meat-rich diet^51^ and a decrease after a 3-month vegetarian diet.^19^ *A. putredinis* has also been positively associated with processed meat intake^53^ and reported at higher fecal abundances in omnivores compared with vegetarians in a large-scale cohort.^15^ Similarly, for *L. lactis*, which is reduced after the flexitarian diet, higher fecal levels have been reported in omnivore versus vegetarian or vegan individuals, and they have been positively associated with dairy intake (*L. lactis*).^15^ Given that *L. lactis* is a lactic acid bacterium commonly present in fermented dairy products, its higher abundance in the control group may directly reflect greater dairy intake. Finally, fecal *B. uniformis,* which decreased after the flexitarian diet, has been previously reported to decrease after a legume-rich diet^54^ and to be positively associated with animal protein intake.^55^

### Shifts in fecal metabolites

Several fecal metabolites related to amino acid metabolism (4-acetamidobutanoic acid, taurine, lysine, carnosine, and diethanolamine) increased after the flexitarian diet. This may reflect enhanced colonic protein and amino acids metabolism due to shifts in microbiota activity and/or increased protein content reaching the colon, possibly due to increased endogenous protein loss associated with fiber intake,^56^ or to the lower digestibility of PP, particularly when consumed as whole foods such as legumes.^57^ Concomitantly, the fecal genetic potential for threonine degradation also increased after the flexitarian diet, in line with the hypothesis of higher endogenous mucin, a protein particularly rich in threonine,^58^ reaching the colon. Conversely, carnitine showed the opposite pattern, with increased levels after the control diet. Even though this metabolite is in its vast majority absorbed in the small intestine, the higher dietary intake (animal products are key dietary sources) could contribute to fecal levels. Supporting this, fecal carnitine levels have previously been associated with meat intake.^59^ Notably, our network analysis revealed a positive correlation between fecal carnitine and *A. putredinis*.

Because animal-based foods (particularly meat and fish) are important purine dietary sources,^60^ the increased fecal levels of purine-related metabolites (e.g. 1-methylxanthine, cytidine, 1-methyluric acid, 1-methyl guanine, adenosine) following the flexitarian diet could not be ascribed to any increase in dietary purine intake and therefore of a greater purine content reaching the colon, unless resulting from a higher fiber intake. Rather, these findings may be explained by alterations in microbial-host purine exchanges, as gut microbiota species generate and metabolize dietary purine, whilst the host excretes and salvages purines.^61^ Consistent with this hypothesis, the genetic potential for the microbial pathway M00580 related to the production of ribose-5-P, the initial substrate for purine synthesis, was increased after the flexitarian diet. Microbially derived purines are known to serve as important substrates for the intestinal mucosa, contributing positively to gut barrier function.^62^

### Increasing plant protein sources modulate microbial tryptophan catabolism toward protective indole propionic acid and kynurenine-related metabolites

Our present findings show that the bacterial tryptophan (TRP) degradation pathways MF0006 and MGB049 were reduced after the flexitarian diet, both pathways reflecting the abundance of the tryptophanase gene (KO1667) responsible for the tryptophan deamination reaction yielding indole production.^63^ These findings reflect the genetic potential of the microbiota to yield this specific metabolic pathway.

In parallel, kynurenic acid and xanthurenic acid levels increased in feces following the flexitarian diet, reflecting host gut and/or microbiota activities. These metabolites originate from the kynurenine pathway, a major route of TRP degradation in mammals that is active in the intestine and can also be mediated by the gut microbiota.^64^ At the circulating level, we previously reported a decrease in indoxyl sulfate (the hepatic conjugate of indole) plasma levels after the flexitarian diet,^22^ while indole propionic acid (IPA) and indole acrylic acid (IAA) plasma levels increased, reflecting different exposures of the host to these particular metabolites. Like indole, IPA and IAA are microbial by-product of TRP catabolism but they are produced by a different microbial enzymatic pathway.^65^ These shifts in microbiota functional potential, fecal metabolite and plasma metabolites suggest together that a PP-rich diet promotes coordinated changes in microbial TRP catabolism, favoring the IPA and kynurenine pathways and resulting in reduced indole production.

Compositional changes in the fecal microbiota also support these shifts in TRP catabolism. Thus, the fecal abundance of species such as *Dorea amylophila* and *A. putredinis*, which decreases with a flexitarian diet, are known to convert TRP into indole.^52^ ^,^ ^66^ Additionally, *Intestinibacter bartlettii* (previously *Clostridium bartlettii*), which contributed to the diet discrimination effect (OPLS-DA model), tended to increase fecal abundance after the flexitarian diet without reaching statistical significance (Wilcoxon), is a known producer of indolelactic acid,^67^ an intermediate metabolite of the IPA pathway. While these compositional shifts are compatible with changes in TRP degradation pathways, a more plausible mechanism may involve the higher fermentable fiber content of the flexitarian diet (36 vs 28 g/d in the CON diet and 21 g/d at baseline), particularly from legumes. Recent *in vitro* and animal studies have reported that the balance between indole and IPA production depends not only on the abundance of TRP-metabolizing bacteria but also on cross-feeding interactions: monosaccharides released from fiber-fermenting bacteria can inhibit tryptophanase activity in indole-producing bacteria, leaving more TRP available for IPA production.^68^ This aligns with evidence that TRP intake correlates with plasma IPA levels only in individuals with both a high prevalence of IPA-producing bacteria and greater fiber intake.^69^

As said before, while only modest differences between FLEX and CON were observed for short-term cardiometabolic outcomes, the present omics analyses revealed distinct shifts in gut microbial TRP metabolism. Importantly, these changes in microbial function may represent early metabolic adaptations that precede measurable clinical benefits and could contribute, over longer-term exposure, to the cardiometabolic protective effects associated with more plant-based dietary patterns.^4-6^ IPA and IAA support gut homeostasis through multiple mechanisms, including activation of the aryl hydrocarbon receptor, which promotes anti-inflammatory IL-22 secretion, enhances tight junction expression, and stimulates GLP-1 release.^63^ Circulating IPA levels have also been inversely associated with type 2 diabetes risk.^69^ ^,^ ^70^ Although indole act as an aryl hydrocarbon receptor agonists with local gut benefits, their hepatic metabolite indoxyl sulfate is a uremic toxin that is prospectively associated with adverse effects on vascular endothelial and smooth muscle cells,^71^ finally resulting in increased risk of cardiovascular diseases.^72^ The health implications of higher fecal kynurenic acid and xanthurenic acid remain uncertain. While circulating kynurenine pathway metabolites have been associated with elevated cardiometabolic risk,^73^ activation of IDO1—the rate-limiting enzyme initiating the kynurenine pathway—in intestinal epithelial cells has been shown to protect against intestinal inflammation, barrier dysfunction, and atherosclerosis in the context of a high-fat diet.^74^ Furthermore, metabolites within this pathway may exert divergent effects: kynurenic acid and xanthurenic acid, in both plasma and feces, have been negatively associated with intestinal inflammation, whereas quinolinic acid has shown a positive association.^75^

### Strengths and limits of the study

This study has several strengths. The effects of increasing PP intake, in the context of a whole-food flexitarian diet, on the fecal microbiome remain poorly characterized, and our findings provide novel insights into the impact of such diets, which are compatible with human and planetary health and may be more acceptable to the general population than vegetarian or vegan diets.^76^ ^,^ ^77^ The randomized cross-over design with strict dietary control establishes a strong causal link between dietary intervention and the observed effects. Furthermore, by characterizing microbiome composition at the species level, assessing functional genetic potential, and integrating fecal and plasma metabolomics, we captured subtle shifts in gut microbial activity that would otherwise have remained undetected. Limitations include the small sample size, the relatively short intervention period compared to lifelong dietary exposure, and limited fecal metabolome coverage. The exclusively male study population also hindered the transposition of these results to females, as both the basal gut microbiota composition^78^ ^,^ ^79^ and the microbiota responses to nutritional interventions have been reported to be sex-specific.^80^ ^,^ ^81^ In conclusion, in men with elevated cardiometabolic risk, increasing PP source intake by adopting a flexitarian diet did not alter overall gut microbiota diversity but induced compositional changes compatible with previous reports and strengthen our understanding of the gut microbiota signature associated with PP-rich diets. We reported shifts in fecal metabolites and genetic functional potential combined with changes in indole metabolites at the circulating level, suggesting that microbial activity was redirected toward the IPA and kynurenine TRP degradation pathways, likely resulting in reduced indole production. According to the existing literature, these changes are expected to favor gut homeostasis and cardiometabolic health. These findings, which provide mechanistic insights into how diets higher in PP sources modulate gut microbiota composition and function, warrant confirmation in larger and longer-term trials including a more diverse population in terms of sex, ethnic background or usual dietary exposure. Additional effort should also be made to directly link the observed shifts in gut TRP catabolism with cardiometabolic outcomes in the context of PP-rich diets.

## Supplementary Material

Supplementary Material Lepine_2026_Gut Microbes_supplemental_review_vf_clean.pdf

## Data Availability

The participants of this study did not give written consent for their data to be shared publicly, so owing to the sensitive nature of the research supporting data is not available.

## Data deposition

NA.

## Abbreviations

CON: control diet
FLEX: flexitarian diet
IAA: indole acrylic acid
IPA: indole propionic acid
MSP: metagenomic species
OPLS-DA: orthogonal-partial least square-discriminant analysis
PP: plant protein
SCFA: short chain fatty acid
