# Supplementary material for: Increasing plant protein sources in the diet modulates gut microbiota and tryptophan metabolism in men at cardiometabolic risk
Source: Gut Microbes. 2026 May 27;18(1):2677951. doi: 10.1080/19490976.2026.2677951 (PMC13217912; doi:10.1080/19490976.2026.2677951)
Supplement: Supplementary Material — Lepine_2026_Gut Microbes_supplemental_review_vf_clean.pdf [file KGMI_A_2677951_SM0684.pdf]

<u>**Supplemental material**</u>

Increasing plant protein sources in the diet modulates gut microbiota and tryptophan metabolism in men at cardiometabolic risk

Authors: Gaïa Lépine, Anne-Marie Davila, Gwendal Cueff, Gisèle Pickering, Farid Ichou, Caroline Perreau, Catherine Lefranc-Millot, Florence Thirion, François Mariotti, Didier Rémond, Hélène Fouillet, Sergio Polakof

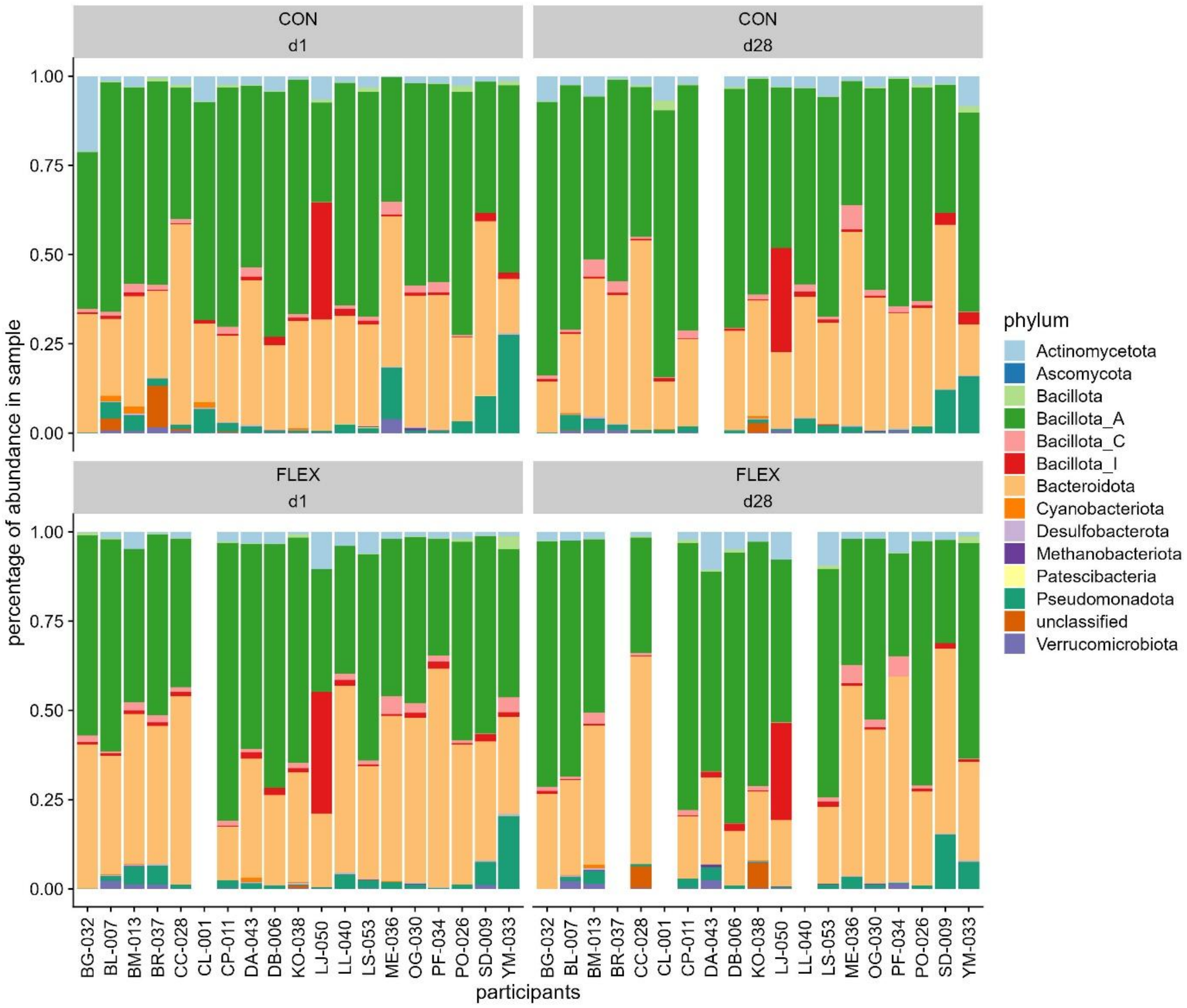

Figure S1: Phylum relative abundance (expressed as percentage) in each participant before (d1) or after (28) a flexitarien (FLEX) and a control (CON) diet, n=19.

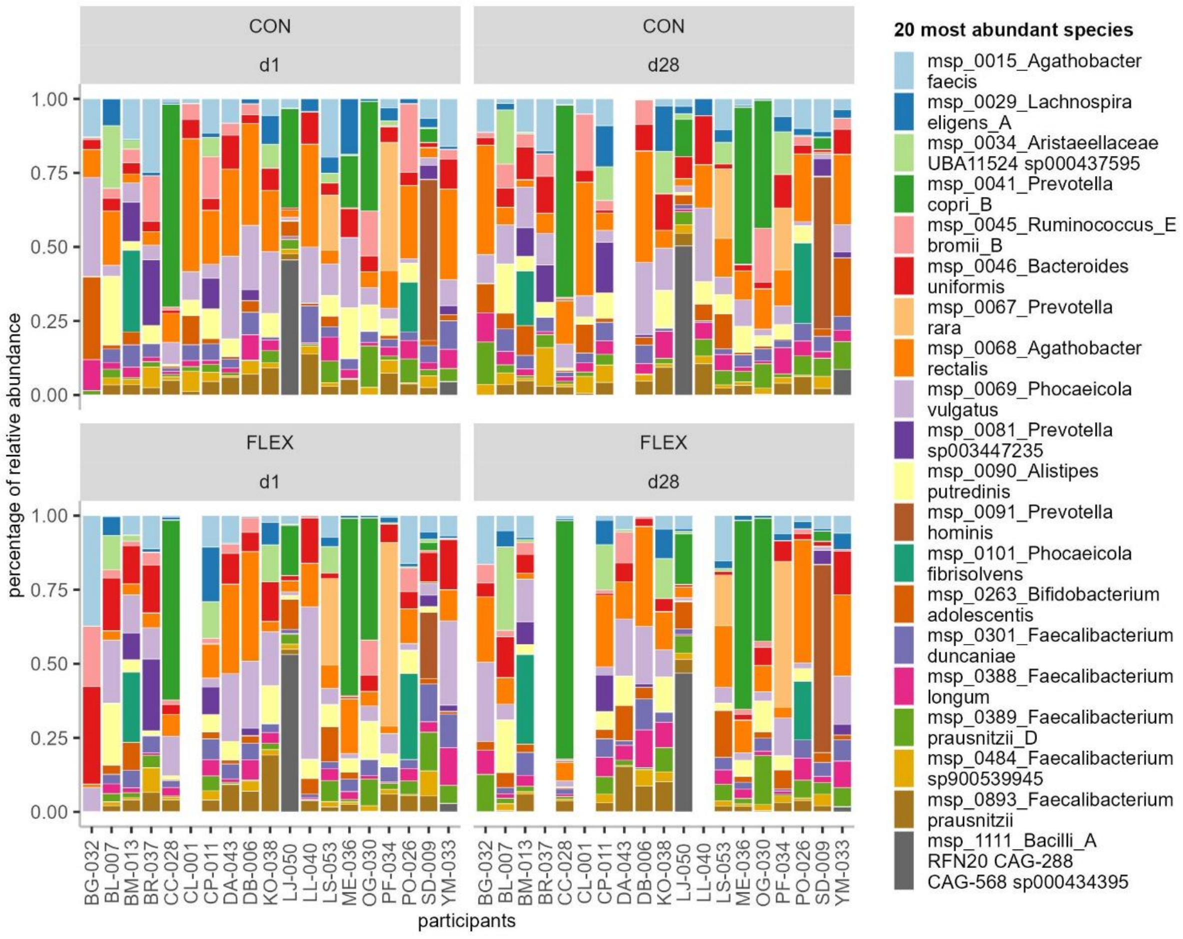

Figure S2: Relative abundance of the top 20 most abundant metagenomics species (expressed as percentage) in each participant before (d1) or after (28) a flexitarien (FLEX) and a control (CON) diet, n=19

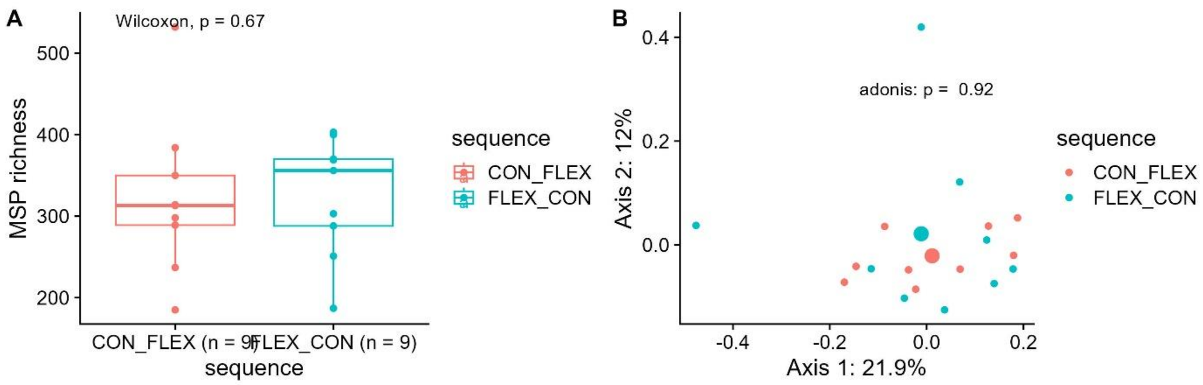

Figure S3: Metagenomic specie richness (alpha diversity) and beta diversity (PCoA) distribution depending on the order diet consumption (control than flexitarian diet (CON_FLEX) or flexitarian than control diet (FLEX_CON), n=19

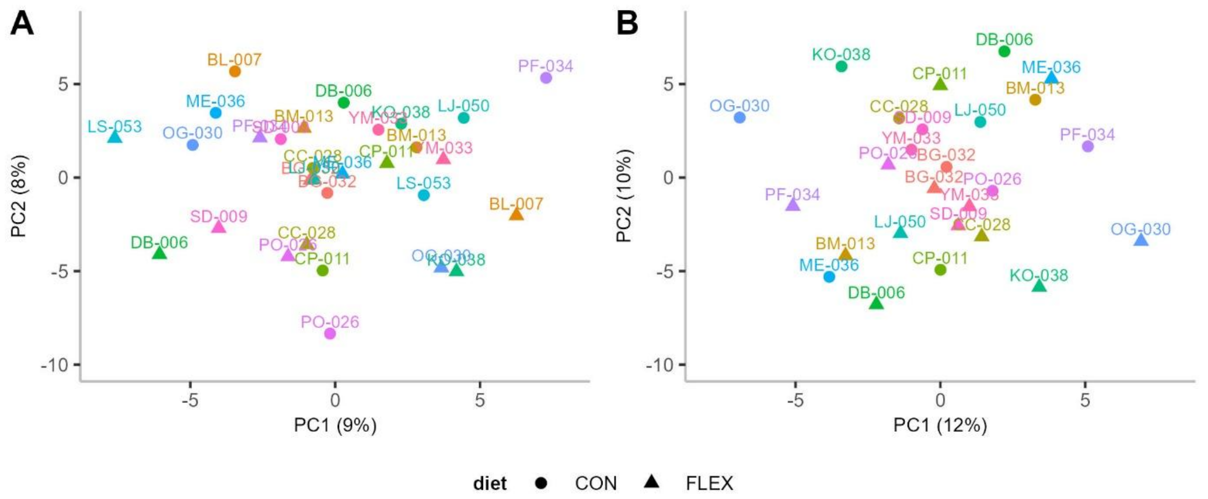

Figure S4: Principal component analysis on fecal metagenomics **species** relative abundance delta (d28-d1) values after a flexitarian and a control diet without (A) or with extraction of only intraindividual variation (B), n=15

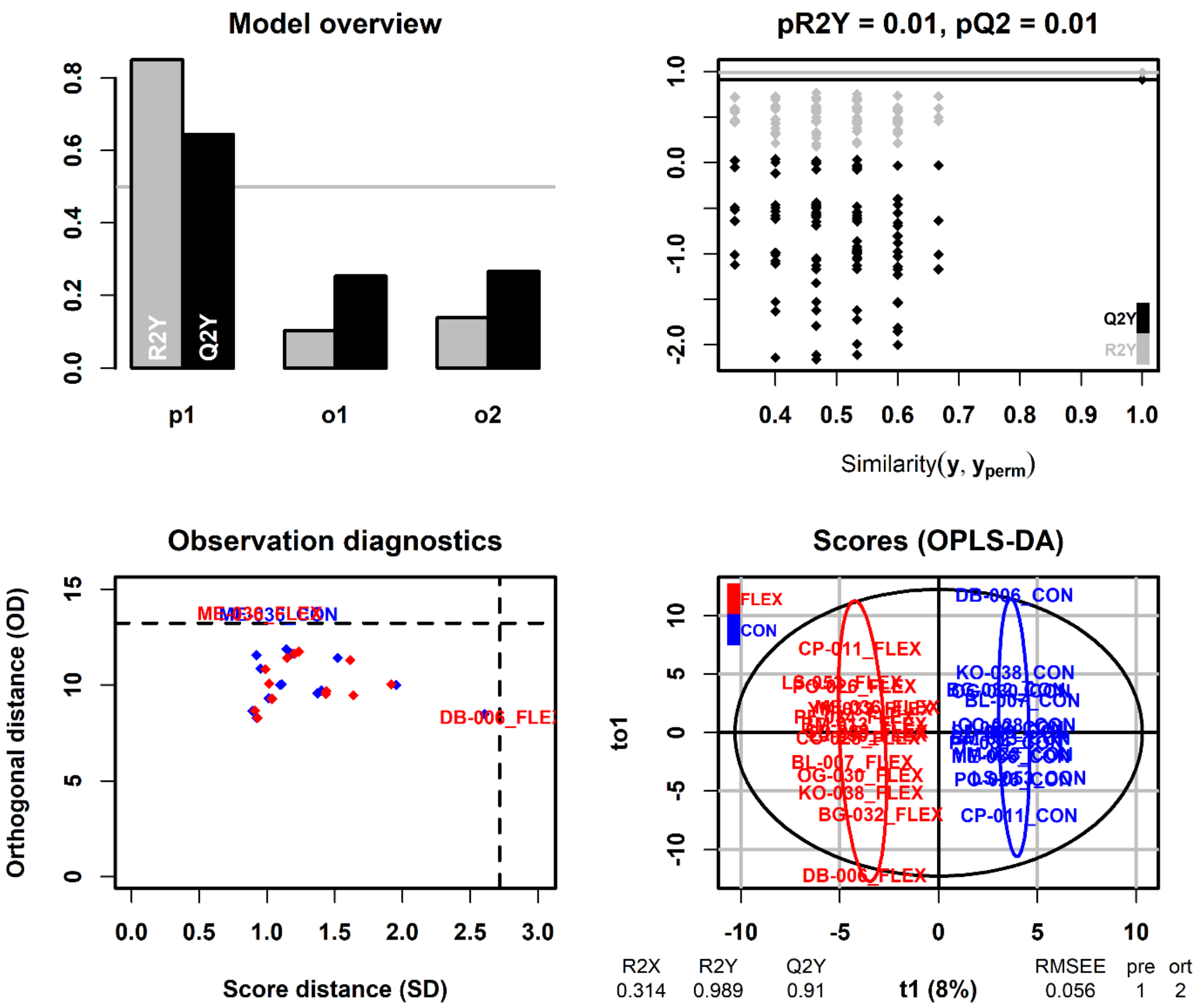

Figure S5: Overview OPLS-DA model performed on fecal metagenomics species relative abundance delta (d28-d1) value after a flexitarian (FLEX) and control omnivore (CON) diet, n=15

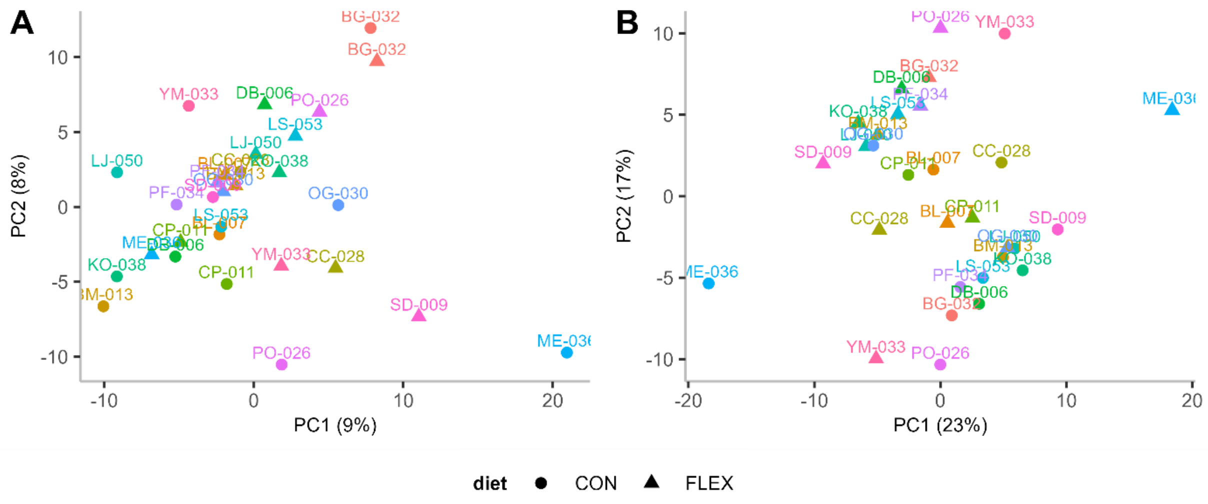

Figure S6: Principal component analysis on fecal metagenomics **pathway** relative abundance delta (d28-d1) values after a flexitarian and a control diet without (A) or with extraction of only intraindividual variation (B), n=15

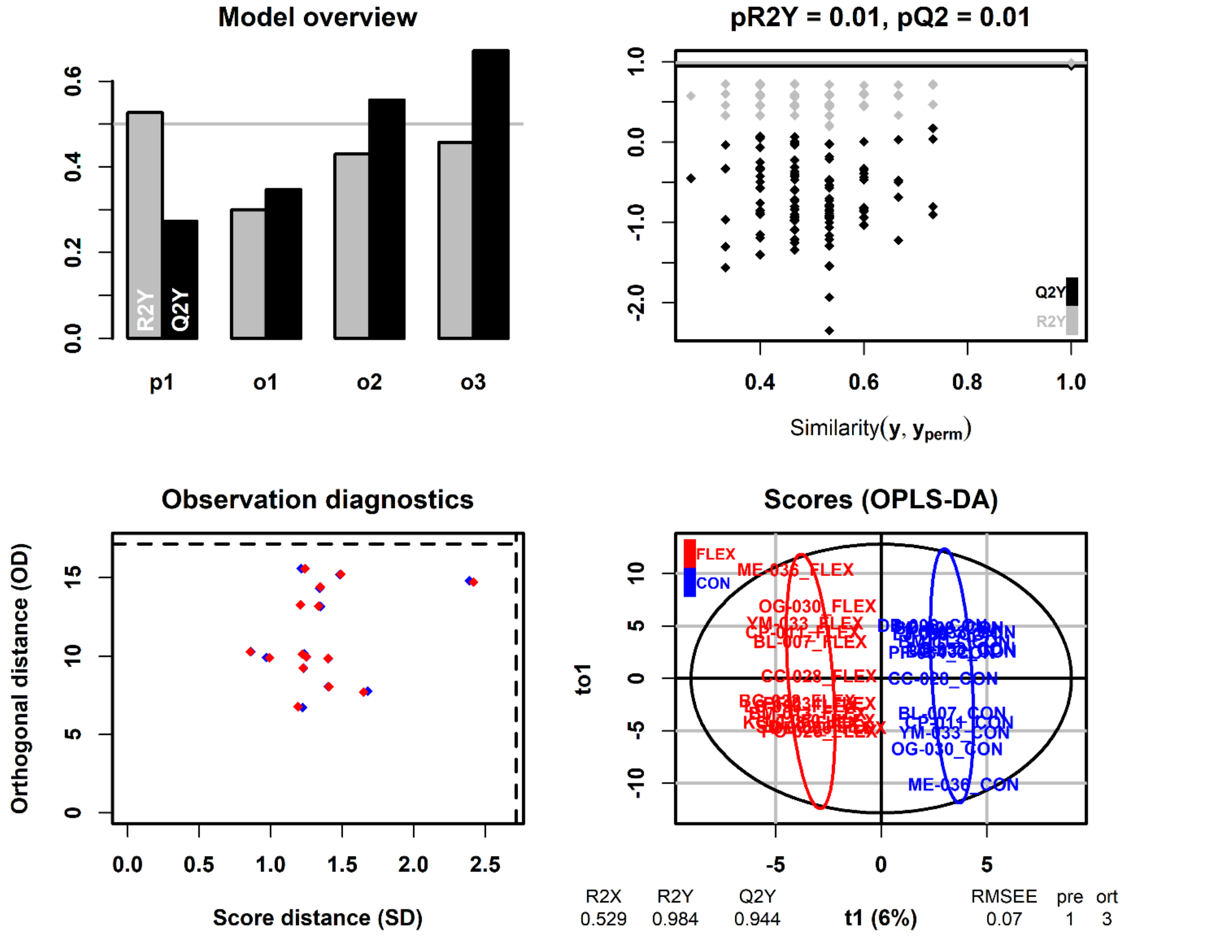

Figure S7: Overview OPLS-DA model performed on fecal metagenomics pathways relative abundance delta (d28-d1) value after a flexitarian (FLEX) and control omnivore (CON) diet, n=15

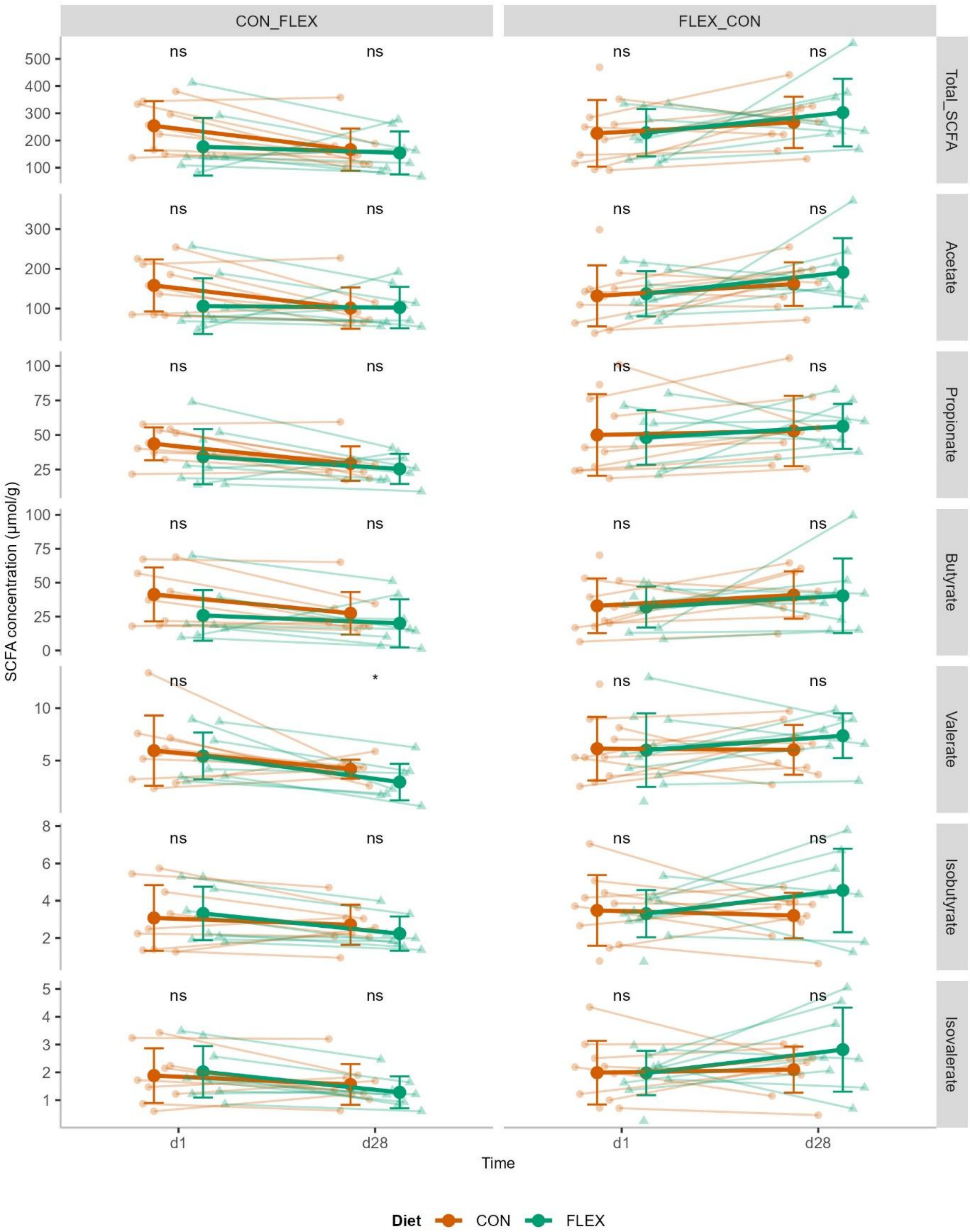

Figure S8: Fecal short-chain fatty acid (SFCA) concentration before (d1) and after (d28) a flexitarian (FLEX) and control omnivore (CON) diet stratified by sequence order (FLEX first (FLEX_CON) or CON first (CON_FLEX)). FLEX and CON values were compared at each timepoint by paired Wilcoxon test. N=19

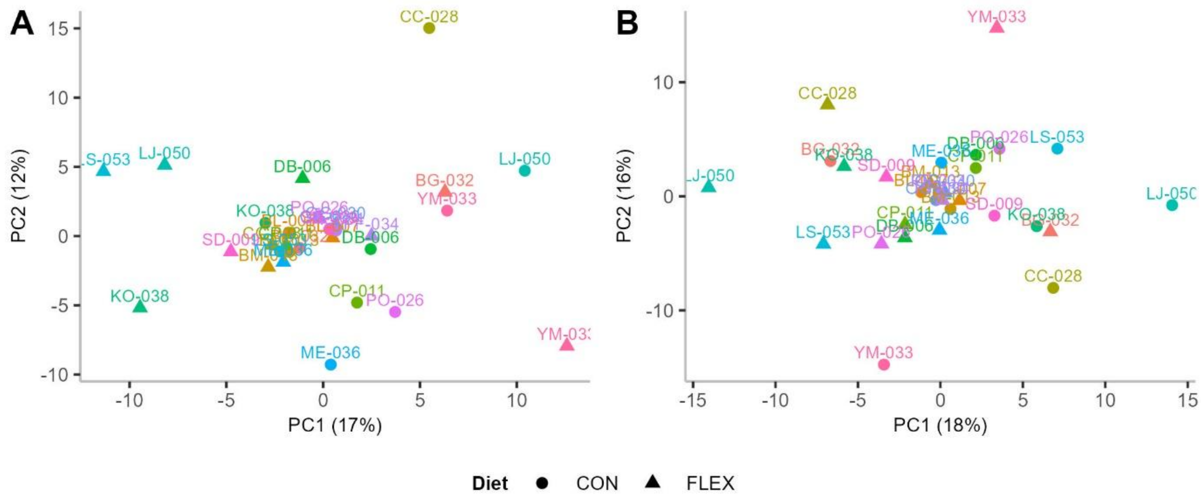

Figure S9: Principal component analysis on fecal **metabolomics** relative abundance delta (d28-d1) values after a flexitarian and a control diet without (A) or with extraction of only intraindividual variation (B), n=15

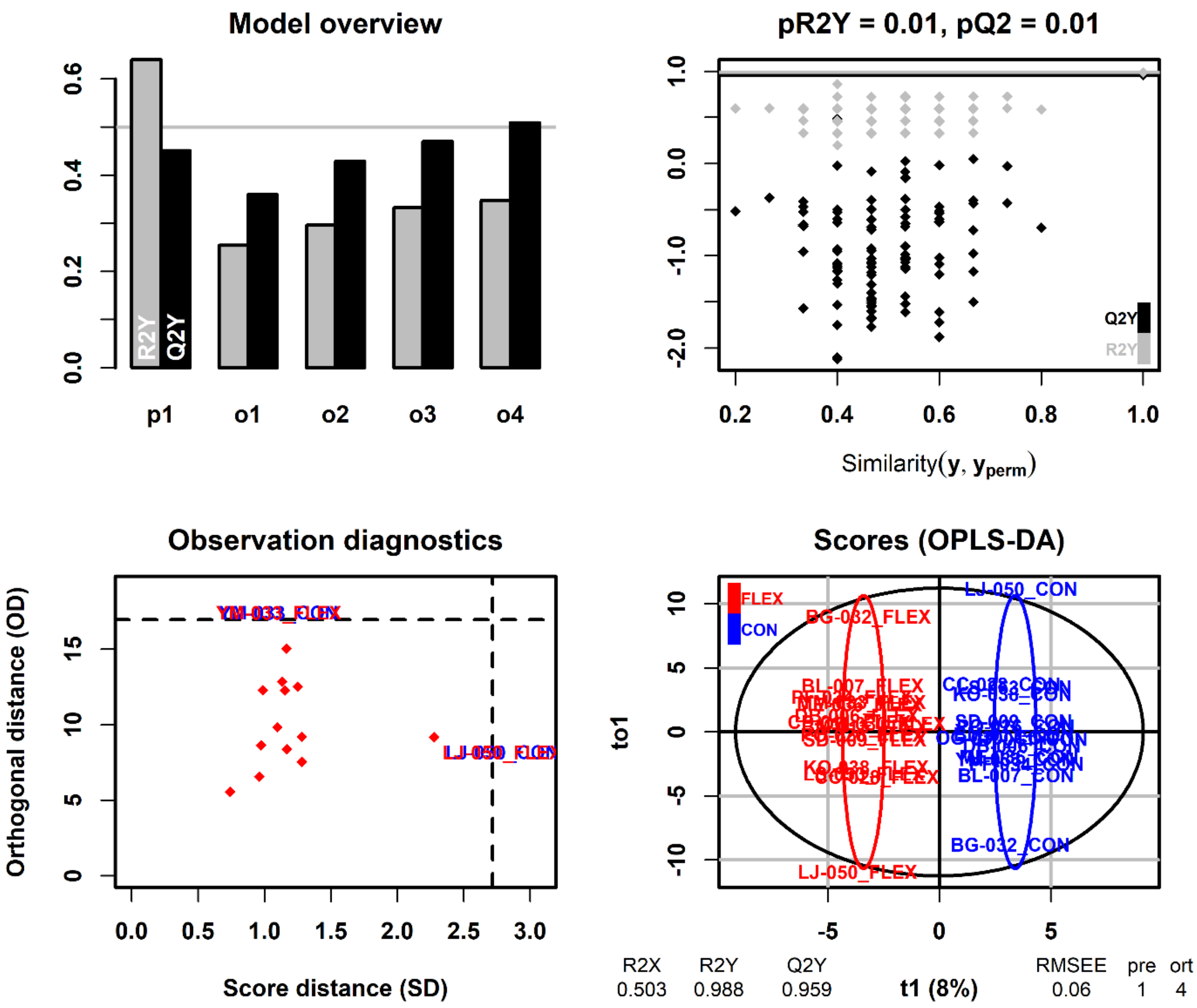

Figure S10: Overview OPLS-DA model performed on fecal metabolomic relative abundance delta (d28-d1) value after a flexitarian (FLEX) and control omnivore (CON) diet, n=15

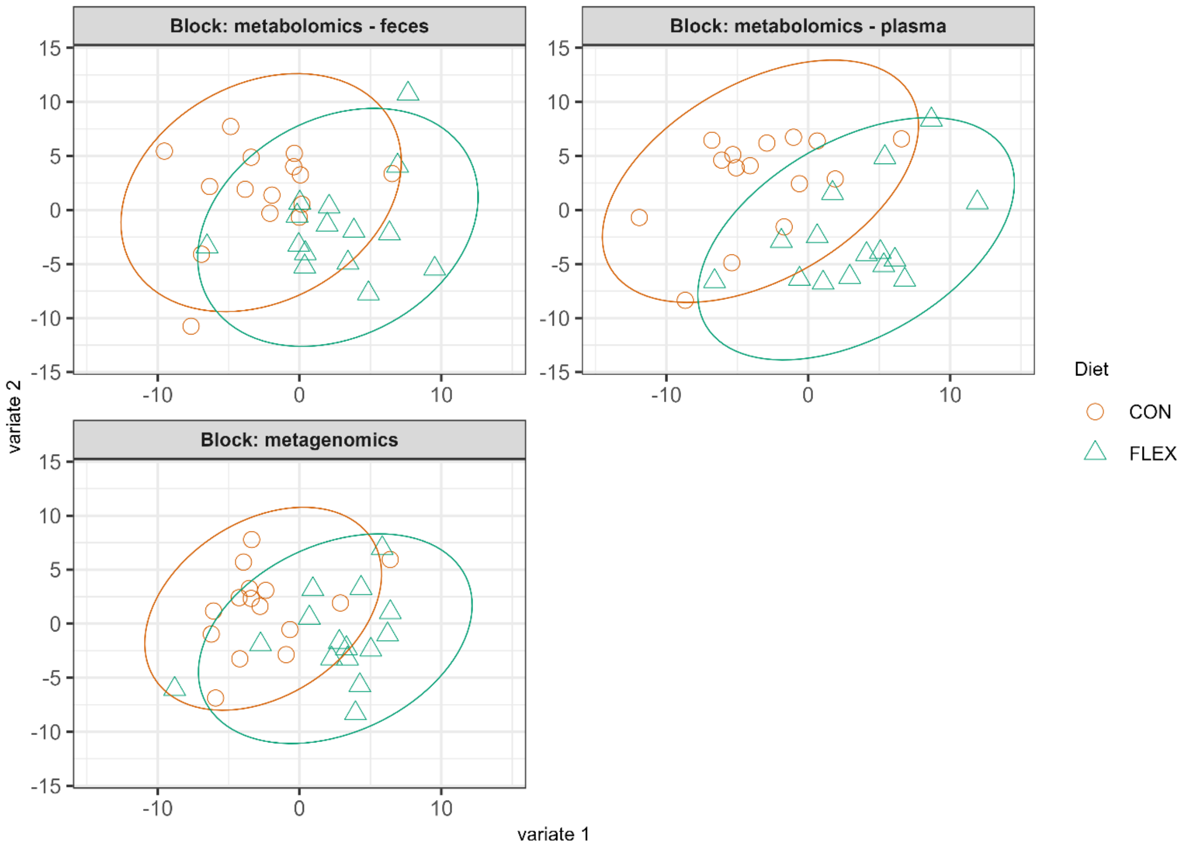

Figure S11: sample separation on principal component 1 and 2 from multiblock PLS-DA performed on fecal metagenomic, fecal metabolome and plasma metabolome delta(d28-d1) values after a flexitarian (FLEX) and a control (CON) diet. Before analysis, data were transformed to extract only within individual variation, n=15

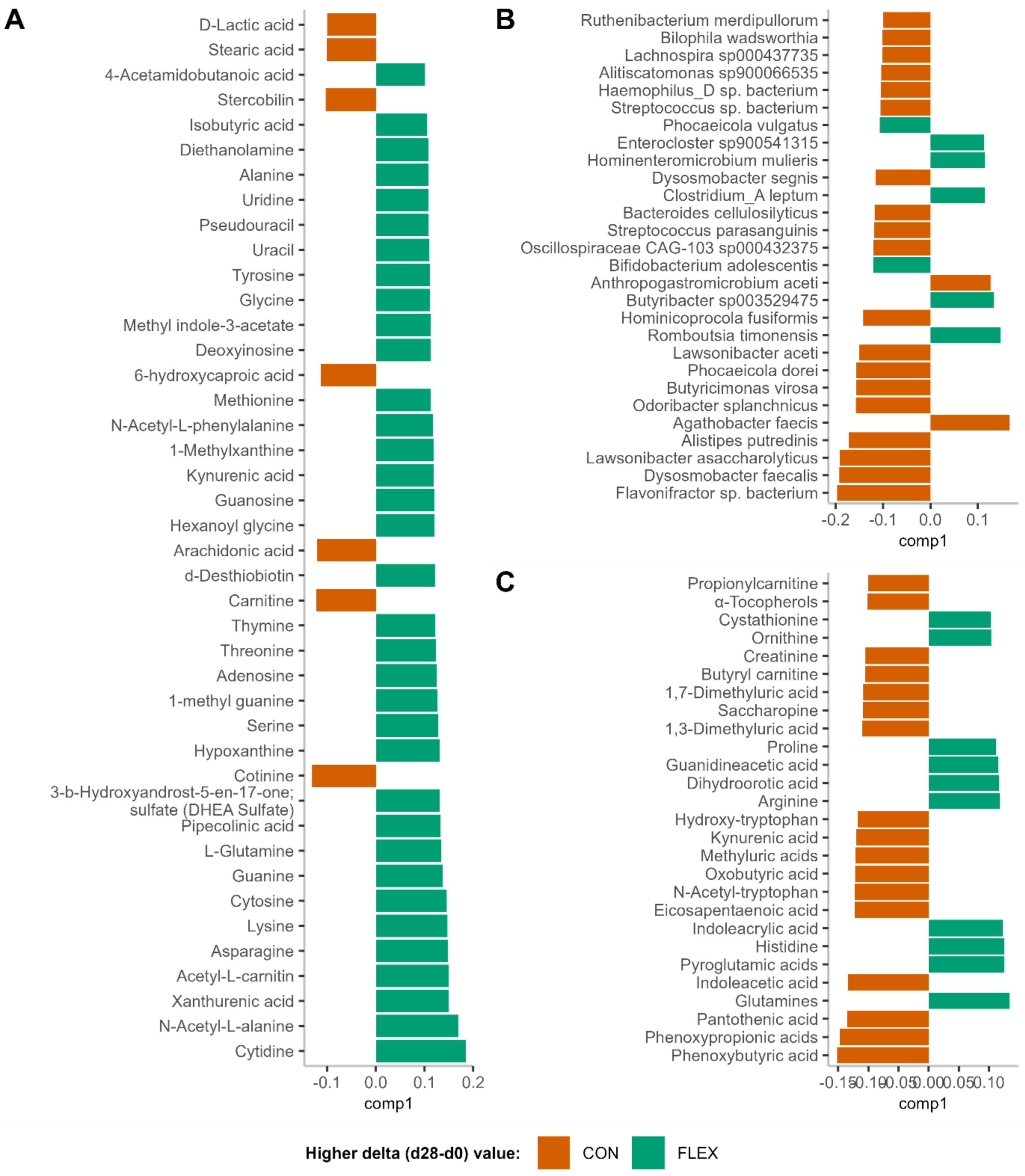

<u>Figure S12:</u> Variables contributing to diet discrimination when comparing delta (d28-d1) value after a flexitarian (FLEX) or control (CON). Features shown have loading score absolute value > 0.1 on principal component 1 of DIABLO model. A: fecal metabolites; B: metagenomic species; C: plasma metabolites, n=15.

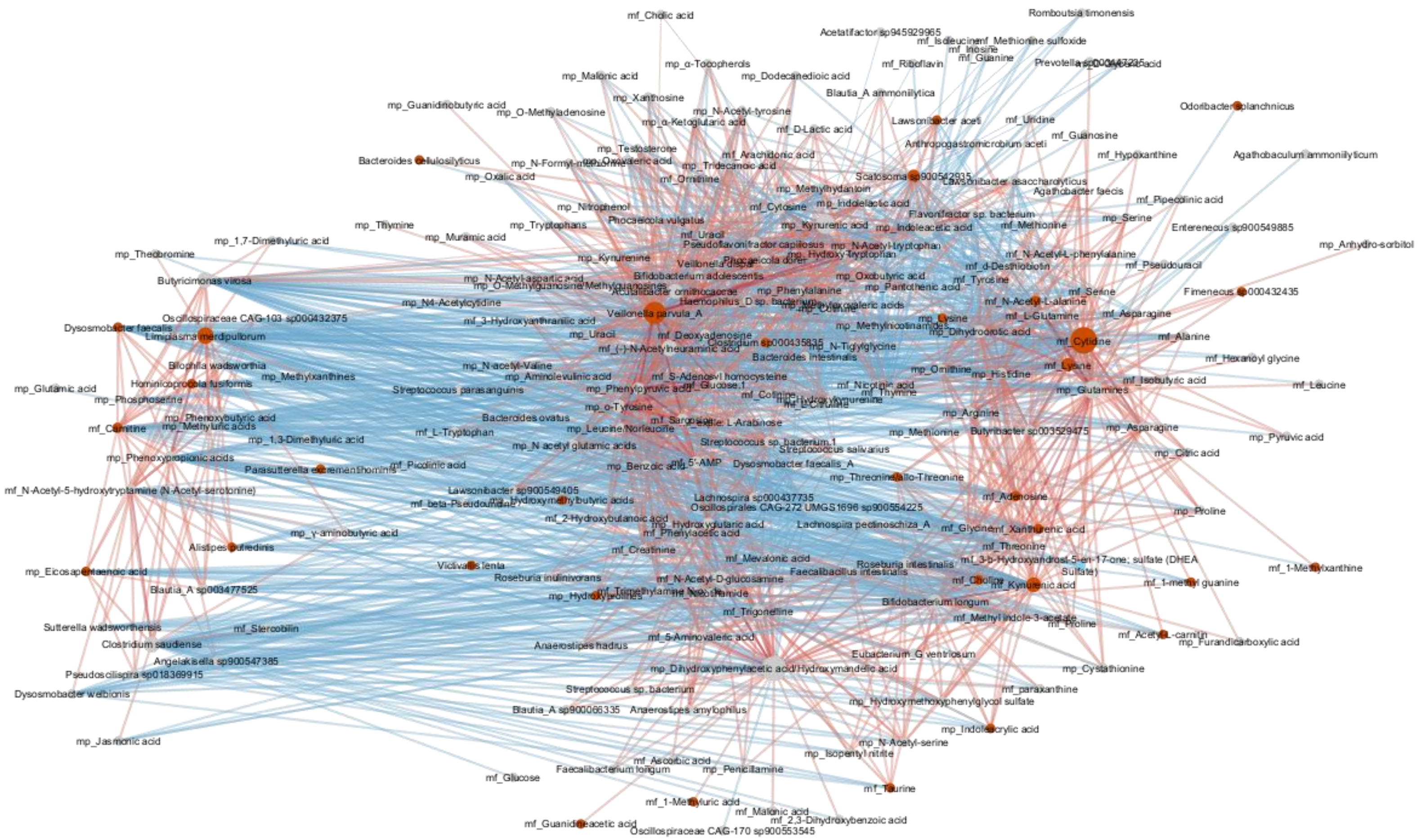

Figure S13: Bipartite relevance network based on multi-bloc PLS-DA analysis representing inter-omics relation between a selection of fecal species, fecal metabolites and plasma metabolites contributing to diet effect. Edges width is proportional to similarity (equivalent to a correlation metric), only relations with a similarity > 0.5 are represented. Edge color is indicative of the direction of the association (red: positive association, blue: negative association). Green node: $\Delta_{d28\text{-}d1}$ FLEX > $\Delta_{d28\text{-}d1}$ CON; Red node: $\Delta_{d28\text{-}d1}$ FLEX < $\Delta_{d28\text{-}d1}$ CON

Supplemental Table S1: Fecal metagenomic species strongly contributing to diet discrimination (Variable Importance in the Projection (VIP) > 1.5), as identified by OPLS-DA on relative abundance delta values ($\Delta_{d28\text{-}d1}$) after the flexitarian (FLEX) and control (CON) diets.

	**Delta data**				**Mean relative abundance delta ($\Delta_{d28\text{-}d1}$) value (sd, n)**	
**species**	**p-wilcoxon[1]**	**Loading[2]**	**VIP**	**Prevalence[3] (%)**	**CON**	**FLEX**
Bacteroides salyersiae	0.109	-0.17	2.32	54	-1.90e-07 (4.26e-07, 7)	4.89e-08 (2.74e-07, 8)
**Dorea_A amylophila**	**0.049***	0.15	2.10	73	-5.70e-08 (4.35e-07, 11)	-2.76e-07 (4.85e-07, 10)
**Alistipes putredinis**	**0.030***	0.15	2.01	99	2.31e-07 (1.86e-06, 15)	-1.10e-06 (1.64e-06, 14)
**Lactococcus lactis**	**0.032***	0.14	1.92	90	5.12e-08 (1.07e-07, 13)	-3.64e-09 (6.42e-08, 12)
Clostridium sp000435835	0.125	-0.14	1.91	55	1.85e-08 (3.35e-08, 6)	4.04e-08 (1.05e-07, 5)
Christensenellales CAG-138 PeH17 sp000435055	0.297	-0.14	1.88	54	-3.77e-08 (2.54e-07, 8)	2.99e-07 (7.66e-07, 8)
**Dysosmobacter segnis**	**0.011***	0.14	1.87	89	3.41e-07 (1.16e-06, 13)	-4.78e-07 (5.67e-07, 13)
**Parasutterella excrementihominis**	**0.037***	0.14	1.85	69	-1.71e-08 (1.94e-07, 10)	-1.39e-07 (2.02e-07, 10)
Suilimivivens sp000438155	0.563	0.14	1.85	55	5.83e-08 (1.00e-07, 8)	-1.85e-08 (5.48e-08, 8)
Oscillospiraceae CAG-170 sp900549635	0.193	-0.13	1.83	68	-7.76e-09 (1.06e-07, 10)	1.22e-07 (2.41e-07, 11)
Methanobrevibacter_A smithii	0.844	-0.13	1.83	49	-1.32e-07 (3.29e-07, 8)	1.70e-08 (1.44e-07, 6)
**Lawsonibacter aceti**	**0.019***	0.13	1.81	90	1.19e-08 (3.12e-08, 13)	-1.61e-08 (4.47e-08, 12)
Limiplasma merdipullorum	0.219	0.13	1.78	54	1.41e-08 (2.33e-08, 8)	-2.56e-08 (4.40e-08, 8)
Scatosoma sp900542935	0.312	-0.13	1.77	32	1.07e-07 (6.89e-07, 6)	6.31e-07 (8.38e-07, 5)
Acutalibacteraceae UBA737 sp900549055	0.109	0.13	1.76	49	2.46e-08 (1.21e-07, 9)	-2.64e-07 (5.13e-07, 8)
Intestinibacter bartlettii	0.102	-0.13	1.72	75	-1.43e-07 (3.73e-07, 11)	4.01e-08 (1.07e-07, 11)
Victivallis lenta	0.098	0.13	1.72	66	3.49e-08 (1.10e-07, 10)	-5.38e-08 (1.39e-07, 10)
**Bacteroides uniformis**	**0.013***	0.13	1.71	100	1.30e-06 (3.35e-06, 15)	-3.23e-06 (5.52e-06, 15)
Otoolea fessa	0.127	0.12	1.66	90	8.18e-08 (3.42e-07, 13)	-2.34e-07 (4.23e-07, 13)
Limivivens sp900066135	0.219	0.12	1.66	49	3.43e-08 (4.95e-08, 7)	-1.32e-08 (2.06e-08, 8)
Dysosmobacter faecalis	0.110	0.12	1.66	96	8.22e-09 (2.30e-08, 14)	-6.87e-09 (2.25e-08, 13)
Fimenecus sp000432435	0.563	-0.12	1.64	58	-5.89e-07 (3.82e-06, 8)	9.87e-07 (1.89e-06, 7)
Hominicoprocola fusiformis	0.080	0.12	1.59	82	3.44e-08 (1.05e-06, 13)	-8.50e-07 (9.55e-07, 13)
Veillonella parvula_A	0.750	0.12	1.58	48	5.22e-08 (1.00e-07, 8)	-6.44e-07 (1.16e-06, 3)
Lachnospiraceae UBA3402 sp003478355	0.233	0.11	1.56	85	-2.39e-08 (6.74e-08, 12)	-6.38e-08 (6.57e-08, 12)
Suilimivivens aceti	0.175	-0.11	1.56	69	-6.36e-09 (3.62e-08, 11)	1.96e-08 (3.22e-08, 12)
Bacteroides cellulosilyticus	0.206	0.11	1.54	80	9.16e-07 (2.68e-06, 11)	-4.47e-07 (1.33e-06, 11)
Odoribacter splanchnicus	0.095	0.11	1.53	100	-5.96e-08 (3.76e-07, 15)	-3.18e-07 (3.63e-07, 15)

1: Relative abundance delta values after CON and FLEX diets were compared by paired Wilcoxon sign-rank test. 2: Loading score in the predictive component (associated to diet discrimination) of the OPLS-DA model. 3:All metrics derived from analyses on 15 participants with all samples available, except fir prevalence which was calculated across all participants (n=19) and associated samples available (n=71).

Supplemental Table S2: Fecal metagenomic pathways strongly contributing to diet discrimination (Variable Importance in the Projection (VIP) > 1.5), as identified by OPLS-DA on relative abundance delta values (Δ_{d28-d1}) after the flexitarian (FLEX) and control (CON) diets, n=15.

						**Mean Δ_{d28-d1} (sd, n)**	
**Code**	**database**	**Biological function / pathway**	**p-wilcoxon[1]**	**Loading score[2]**	**VIP**	**CON**	**FLEX**
MGB053	GBM	**Butyrate synthesis II**	**0.013***	0.19	2.61	1.73e-07 (1.90e-06, 15)	-1.47e-06 (1.92e-06, 15)
MF0116	GMM	**butyrate production via transferase**	**0.026***	0.18	2.53	1.74e-07 (1.91e-06, 15)	-1.42e-06 (1.87e-06, 15)
MF0099	GMM	methanol conversion	0.578	-0.17	2.35	-1.99e-07 (3.77e-07, 10)	1.26e-07 (2.29e-07, 8)
M00365	KEGG	**C10-C20 isoprenoid biosynthesis, archaea**	**0.007****	0.17	2.33	2.26e-06 (5.26e-06, 15)	-4.68e-06 (6.74e-06, 15)
MF0009	GMM	**tryptophan degradation**	**0.026***	0.16	2.17	5.06e-07 (1.15e-05, 15)	-9.80e-06 (1.19e-05, 15)
M00580	KEGG	Pentose phosphate pathway, archaea, fructose 6P => ribose 5P	0.054	-0.15	2.08	-2.00e-07 (4.06e-07, 12)	7.42e-07 (1.77e-06, 13)
MF0030	GMM	**threonine degradation (formate pathway)**	**0.048***	-0.14	1.97	1.12e-06 (2.02e-05, 15)	2.14e-05 (2.51e-05, 15)
MF0049	GMM	maltose degradation	0.135	0.14	1.93	3.08e-06 (5.62e-06, 15)	-3.13e-07 (2.58e-06, 15)
MF0048	GMM	**lactose degradation**	**0.048***	-0.13	1.83	1.98e-06 (1.16e-05, 15)	1.10e-05 (1.27e-05, 15)
M00159	KEGG	V/A-type ATPase, prokaryotes	0.151	-0.13	1.82	-1.86e-06 (2.93e-06, 15)	1.89e-07 (2.40e-06, 15)
MF0050	GMM	melibiose degradation	0.135	-0.12	1.71	1.33e-06 (1.11e-05, 15)	9.76e-06 (1.38e-05, 15)
M00652	KEGG	Vancomycin resistance, D-Ala-D-Ser type	0.084	0.12	1.69	5.99e-07 (1.89e-06, 12)	-9.70e-07 (1.39e-06, 10)
M00002	KEGG	Glycolysis, core module involving three-carbon compounds	0.107	-0.12	1.68	-4.66e-06 (1.36e-05, 15)	5.98e-06 (1.46e-05, 15)
M00549	KEGG	Nucleotide sugar biosynthesis, glucose => UDP-glucose	0.135	-0.12	1.65	-3.00e-06 (1.15e-05, 15)	4.44e-06 (9.31e-06, 15)
M00098	KEGG	Acylglycerol degradation	0.151	0.12	1.64	2.66e-07 (1.02e-06, 15)	-4.33e-07 (8.59e-07, 15)
MF0111	GMM	triacylglycerol degradation	0.151	0.12	1.64	2.66e-07 (1.02e-06, 15)	-4.33e-07 (8.59e-07, 15)
MF0085	GMM	pyruvate:formate lyase	0.073	-0.12	1.64	4.03e-06 (1.56e-05, 15)	1.44e-05 (1.62e-05, 15)
MF0052	GMM	chondroitin sulfate and dermatan sulfate degradation	0.121	0.12	1.62	6.13e-07 (8.43e-06, 15)	-6.14e-06 (1.00e-05, 15)
M00570	KEGG	Isoleucine biosynthesis, threonine => 2-oxobutanoate => isoleucine	0.083	-0.12	1.60	2.09e-06 (2.06e-05, 15)	1.74e-05 (2.22e-05, 15)
MF0074	GMM	mannitol degradation	0.208	-0.11	1.57	-1.24e-06 (4.32e-06, 15)	1.35e-06 (4.62e-06, 15)
MGB049	GBM	**Tryptophan degradation**	**0.048***	0.11	1.54	-1.30e-07 (1.65e-05, 15)	-1.00e-05 (1.24e-05, 15)
MF0053	GMM	allose degradation	0.599	-0.11	1.53	-1.03e-06 (3.48e-06, 15)	5.12e-07 (1.32e-06, 15)
M00082	KEGG	Fatty acid biosynthesis, initiation	0.107	-0.11	1.53	-5.11e-06 (1.15e-05, 15)	2.84e-06 (1.18e-05, 15)
M00083	KEGG	Fatty acid biosynthesis, elongation	0.083	-0.11	1.52	-5.34e-07 (1.55e-05, 15)	8.96e-06 (1.69e-05, 15)

1: p-value associated to paired Wilcoxon sign-rank test comparing Δ_{d28-d1} values after CON and FLEX diets. 2: Loading score in the predictive component (associated to diet discrimination) of the OPLS-DA model

Supplemental table S3: Short-chain fatty acid concentration in dry fecal matter before and 28 days after a control (CON) and a flexitarian (FLEX) diets. Effect of diet randomization order (seq), diet, time and their interaction were assessed using Wald test statistics.

	CON diet		FLEX diet		p-values						
	**d1**[1] N = 19	**d28**[1] N = 18[1]	**d1**[1] N = 19[1]	**d28**[1] N = 16[1]	**seq**	**diet**	**time**	**Seq* diet**	**Diet* time**	**Seq* time**	**Seq* time* diet**
Acetate (µmol/g)	144 (71)	131 (61)	122 (64)	147 (83)	0.031*	0.555	0.692	0.093	0.257	0.003**	0.231
Propionate (µmol/g)	47 (23)	41 (23)	42 (21)	41 (21)	0.003**	0.620	0.301	0.100	0.355	0.000***	0.775
Butyrate (µmol/g)	37 (20)	34 (18)	29 (17)	30 (25)	0.229	0.050	0.560	0.181	0.699	0.020*	0.298
Isobutyrate (µmol/g)	3.29 (1.79)	2.96 (1.15)	3.31 (1.31)	3.39 (2.04)	0.159	0.643	0.354	0.364	0.987	0.009**	0.141
Valerate (µmol/g)	6.05 (3.10)	5.10 (1.99)	5.73 (2.91)	5.16 (2.96)	0.008**	0.824	0.347	0.210	0.892	0.005**	0.127
Isovalerate (µmol/g)	1.94 (1.04)	1.83 (0.81)	2.00 (0.84)	2.05 (1.36)	0.091	0.731	0.311	0.407	0.679	0.000***	0.385
Total_SCFA ((µmol/g)	239 (107)	216 (99)	204 (97)	228 (126)	0.025*	0.478	0.972	0.100	0.199	0.002**	0.387

[1]Mean (SD)

Supplemental Table S4: Fecal metabolites strongly contributing to diet discrimination (Variable Importance in the Projection (VIP) > 1.5), as assessed by OPLS-DA on relative abundance delta values (Δ_{d28-d1}) after the flexitarian (FLEX) and control (CON) diets, n=15.

				**Delta data**			**Mean Δ_{d28-d1} (sd, n)**	
**Metabolite**	**m/z[1]**	**RT[2]**	**Id level[2]**	**p-wilcoxon[4]**	**Loading[5]**	**VIP**	**CON (n=15)**	**FLEX (n=15)**
**4-Acetamidobutanoic acid**	144.0666	3.94	2	**0.005**	-0.20	2.47	-9.96e+06 (1.14e+07)	2.06e+06 (1.10e+07)
1-Methylxanthine	167.0564	3.59	2	**0.015**	-0.18	2.26	-1.14e+08 (1.64e+08)	6.12e+07 (2.04e+08)
Cytidine	244.0927	5.35	2	**0.010**	-0.18	2.25	-8.29e+07 (1.61e+08)	6.91e+07 (2.12e+08)
N-Acetyl-L-alanine	130.0508	3.87	1	**0.013**	-0.18	2.21	-1.23e+07 (3.51e+07)	3.12e+06 (3.29e+07)
1-Methyluric acid	181.0362	4.66	1	**0.026**	-0.17	2.17	-2.73e+08 (3.60e+08)	-2.58e+07 (4.61e+08)
Taurine	124.0072	6.91	1	**0.035**	-0.17	2.08	-5.40e+08 (9.61e+08)	3.40e+08 (1.08e+09)
Xanthurenic acid	206.0449	6.84	2	**0.013**	-0.15	1.92	-5.27e+06 (3.45e+07)	4.21e+07 (7.19e+07)
Kynurenic acid	190.0499	2.24	1	0.064	-0.15	1.91	-1.04e+07 (7.61e+07)	5.64e+07 (9.83e+07)
1-methyl guanine	166.0725	3.65	2	0.083	-0.15	1.88	-1.31e+08 (5.51e+08)	3.31e+08 (6.15e+08)
Carnitine	162.1125	6.89	1	**0.048**	0.14	1.81	1.29e+08 (3.30e+08)	-1.56e+08 (3.19e+08)
Lysine	147.1128	10.28	1	**0.048**	-0.14	1.71	-2.85e+07 (5.62e+07)	5.26e+06 (2.37e+07)
Hippuric acid	180.0655	2.68	1	0.095	-0.13	1.68	-1.84e+07 (2.94e+07)	4.71e+06 (3.57e+07)
Adenosine	268.1041	3.11	2	0.188	-0.13	1.60	-8.23e+07 (3.48e+08)	1.43e+08 (3.80e+08)
Acetyl-L-carnitin	204.1231	3.06	1	**0.048**	-0.13	1.58	-5.27e+07 (9.71e+07)	7.43e+06 (5.71e+07)
Carnosine	227.1139	7.22	2	0.330	-0.12	1.56	-8.25e+07 (2.42e+08)	1.93e+07 (9.15e+07)
Guanidineacetic acid	118.0612	7.72	2	0.073	-0.12	1.56	-1.92e+07 (5.79e+07)	4.29e+07 (1.03e+08)
6-hydroxycaproic acid	131.0713	3.37	2	0.188	0.12	1.55	3.04e+07 (4.29e+08)	-1.54e+08 (2.06e+08)
Diethanolamine	106.0863	5.82	2	0.303	-0.12	1.54	-6.81e+05 (6.93e+06)	3.03e+06 (8.66e+06)
Choline	104.1070	10.01	1	0.229	-0.12	1.51	-7.55e+08 (1.89e+09)	6.22e+08 (2.70e+09)

1: mass to charge ratio; 2: retention time; 3: identification level (1 = fully identified metabolites; 2 = putatively annotated metabolites); 4: p-value from the paired Wilcoxon signed-rank test comparing Δ_{d28-d1} values between CON and FLEX diets; 5: loading score in the predictive component (associated with diet discrimination) of the OPLS-DA model.
